# 3-Monothiopomalidomide, a new immunomodulatory imide drug (IMiD), blunts inflammation and mitigates ischemic stroke in the rat

**DOI:** 10.1007/s11357-025-01573-1

**Published:** 2025-03-17

**Authors:** Kai-Yun Chen, Shih-Chang Hsueh, Pathik Parekh, Buyandelger Batsaikhan, David Tweedie, Weiming Luo, Chirag Patel, Yung-Hsiao Chiang, Nicholas Bambakidis, Barry J. Hoffer, Chi-Zong Huang, Seong-Jin Yu, Kuo-Jen Wu, Yun Wang, Eunji Hong, Dong Seok Kim, Nigel H. Greig

**Affiliations:** 1 Ph.D. Program in Medical Neuroscience, College of Medical Science and Technology, Taipei Medical University, Taipei, 110 Taiwan; 2 Neuroscience Research Center, Taipei Medical University, Taipei, 110 Taiwan; 3 Drug Design & Development Section, Translational Gerontology Branch, Intramural Research Program National Institute on Aging, NIH, Baltimore, MD 21224 USA; 4 Department of Neurosurgery, Department of Surgery, School of Medicine, College of Medicine, Taipei Medical University, Taipei, 110 Taiwan; 5 Department of Neurosurgery, University Hospitals of Cleveland, Cleveland, OH 44106 USA; 6 Center for Neuropsychiatric Research, National Health Research Institutes, Zhunan, 35053 Taiwan; 7 School of Pharmacy, College of Pharmacy, China Medical University, Taichung, Taiwan; 8 Aevis Bio Inc, Daejeon, 34141 Republic of Korea; 9 Aevisbio Inc, Gaithersburg, MD 20878 USA

**Keywords:** Neuroinflammation, Pomalidomide, Ischemic stroke, Immunomodulatory imide drugs, Lipopolysaccharide

## Abstract

An overactive neuroinflammatory response is often evident in the elderly and is a significant contributor to brain tissue damage following acute ischemic stroke. Such an inflammatory response is largely mediated by microglial cells and peripheral blood mononuclear cells (PBMCs). Classical anti-inflammatory agents have not proved clinically effective in mitigating the impact of ischemic stroke but have highlighted targets for new drug development, in particular excessive proinflammatory cytokine release. The immunomodulatory imide drug (IMiD) class has shown potential in reducing neuroinflammation and switching microglial phenotypic expression away from a proinflammatory to a regenerative anti-inflammatory one. 3-Monothiopomalidomide (3-MP), a new IMiD, has a brain/plasma concentration ratio of 0.5 to 0.6, an oral bioavailability of 38.5%, and a monophasic disappearance of half-life 3.2 h following oral administration. 3-MP pretreatment mitigates lipopolysaccharide (LPS)-induced inflammation in cellular human PBMCs and, in rat studies, 3-MP pretreatment lowers proinflammatory cytokine levels in the conditioned media and in plasma and the brain, respectively. Administered systemically to rats challenged with middle cerebral artery occlusion (MCAo) and reperfusion, 3-MP post-MCAo treatment reduced infarction volume; improved body asymmetry, a behavioral measure of stroke impact; and lowered inflammation. In summary, 3-MP exerted neuroprotective effects via anti-inflammatory actions against MCAo-induced ischemic injury and represents a therapeutic that warrants further investigation as a treatment for brain damage and related disorders associated with excessive inflammation.

**Supplementary Information:**

The online version contains supplementary material available at 10.1007/s11357-025-01573-1.

## Introduction

Stroke represents the third major cause of morbidity and mortality in many developed countries, with the preponderance being ischemic and hemorrhage stroke. Of these, ischemic stroke accounts for some 80% of all strokes in the US. In contrast, hemorrhagic stroke represents 10 to 15% of all strokes, albeit its prevalence is higher in Asia [1, 2]. As a consequence, the primary focus of stroke drug trials has been on ischemic strokes [2–4], as survivors routinely experience long-term neurological complications. In this regard, aging is the greatest non-modifiable stroke risk factor, with approximately 75% of all strokes occurring in persons aged ≥ 65 years. This complicates stroke drug trials, and with the population aged ≥ 65 years projected to rise, the number of older adults with incident strokes is expected to increase [6].

The neurochemical/neurobiological changes that are elicited by focal cerebral ischemia are termed the ischemic cascade. This entails cellular bioenergetic failure attributable to cerebral hypoperfusion, oxidative stress, excitotoxicity, microvascular injury, and blood–brain barrier (BBB) disruption together with post-ischemic inflammation. These, combined, result in the dysfunction and death of neurons, glia, and endothelial cells [7, 8].

The inflammatory cascade represents a double-edged sword that, depending on its level of activation and time-dependence following tissue injury, may prove beneficial by activating neuroprotective pathways or deleterious by exacerbating cellular dysfunction and inhibiting homeostatic mechanisms [9, 10]. The inflammatory response encompasses multiple diverse immune cells, pro- and anti-inflammatory mediators, and extracellular receptors. In particular, microglial and astrocytic cells rapidly sense and react to ischemia-induced reactive oxygen and nitrogen species (ROS, RNS), which trigger their generation and the secretion of inflammatory cascade factors that include cytokines, chemokines, and inducible nitric oxide synthase (iNOS) [3, 4, 10]. Amongst the key cytokines released within the brain after ischemia are tumor necrosis factor-α (TNF-α) and interleukin (IL)−1, IL-6, and IL-10 [3, 4, 9, 11–14]. Whereas IL-1 is a pro-inflammatory cytokine, and IL-6 has a largely proinflammatory role in the brain, TNF-α has multiple functions and can potentially influence cell death and/or survival via different pathways and is hence considered a master regulator of the inflammatory response [3, 4, 9, 11]. Elevations in proinflammatory cytokines and reductions in anti-inflammatory IL-10 correlate with larger infarction volumes and worse clinical outcomes following ischemic stroke [15]. Achieving and maintaining the appropriate time-dependent balance between pro- and anti-inflammatory mediators is hence critical in motivating brain healing and optimally promoting regenerative processes, particularly in aging which often is accompanied with impairments in the regulation and function of the immune system potentially impacting both the innate and adaptive responses [4, 5, 11–14]

Despite promising results in experimental studies, inflammation-ameliorating treatments have not yet successfully translated to the clinical setting [14]. In this regard, non-steroidal anti-inflammatory drugs (NSAIDs), for example, represent one of the most effective and widely used anti-inflammatory drug classes. However, an increased risk of stroke associated with their use [16, 17] precludes them as a treatment strategy for ischemic stroke. On evaluating less conventional and potentially more innovative therapeutic strategies possessing inflammation ameliorating actions, the immunomodulatory imide drug (IMiD) class notably lowers the generation of proinflammatory cytokines. Thalidomide and its second- and third-generation analogues, lenalidomide and pomalidomide, are clinically approved IMiDs used in the treatment of multiple myeloma [18, 19] as well as in Hansen’s disease [20, 21] that is associated with a significant inflammatory element [22].

IMiDs have recently demonstrated efficacy in preclinical models of neurological disorders possessing an inflammatory and neuroinflammatory component [23–27]. On this basis, using a phenotypic drug discovery approach [28], the new IMID 3,6’-dithiopomalidomide (3,6’-DP) was developed to mitigate inflammation. 3,6’-DP proved particularly efficacious when administered in cell culture and across animal models of neurodegeneration (TBI, AD, and stroke) [26, 29–32].

Our recent pharmacokinetic evaluations in rats, however, determined that 3,6’-DP disappears relatively rapidly from plasma and has low oral bioavailability — factors that could potentially limit an agent’s clinical development as an oral drug [33]. We therefore sought a more metabolically stable close analogue that retains cellular and in vivo anti-inflammatory action and focused on the agent 3-monothiopomalidomide (3-MP). In the current study, 3-MP was evaluated as a new IMiD to mitigate elevations in proinflammatory cytokines in cellular and animal models and was then appraised in the middle cerebral artery occlusion (MCAo) model of ischemic stroke in rats. MCAo induces neuronal damage within the striatum and cortex and triggers inflammatory processes [34]. Immunomodulation of this over-excessive inflammatory response and ensuing neuronal death provides a therapeutic strategy to facilitate neuroprotection against cerebral ischemic injury.

## Materials and methods

### A. Drug synthesis and preparation

3-MP, an orange-red powder whose structure was confirmed by chemical characterization, was provided by Aevis Bio Inc. (Republic of Korea).

#### 3-MonothioPomalidomiode nanosuspension

Thalidomide, pomalidomide, and most related analogues have poor aqueous solubility. As a consequence, to aid biological evaluation, a 3-MP nanosuspension was prepared in line with our prior study [35]. Specifically, the drug was mixed in an aqueous solution (Tween 80/water) and vortexed for 10 min. The coarse drug suspension was then placed in Eppendorf microtubes containing approximately 0.4 g of 0.1–0.2 mm yttrium-stabilized zirconia-silica beads (Silibeads® Typ ZY Sigmund Lindner, Germany). These were vortexed twice, at 3000 rpm for 45 min, using a beads-milling cell disruptor (Disruptor Genie®, Scientific Industries, USA). The resulting nanosuspension was collected from each microtube and sieved through a 40-µm filter (Cell Strainer, Falcon no. 352340) to remove the zirconia-silica beads. Essentially, 87.6 mg of the drug was prepared in Tween-80 (60 µl) and made up to a final volume of 8 ml with water (87.6 mg/60 µl/7853 µl). The stock drug concentration was 10.83 mg/ml (37.4 mM). The drug “vehicle” was the same ratio of Tween 80 and water, processed in the exact same way as the drug preparation (but without the inclusion of drug). To evaluate the stability of the 3-MP nanosuspension, a vial (241 μM, 4 °C) was time-dependently sampled over a 98-day period, and the drug was quantified by LC–MS (see Supplementary information).

### B. Cellular studies

Human peripheral blood mononuclear cells (PBMCs), purchased from ATCC (Cat no. PCS-800–011, Lot no. 81215231, Manassas, VA, USA), were washed with ice-cold HBSS (ATCC 30–2213) containing 10% fetal bovine serum (FBS: ATCC 30–2020) and used for the experiment directly. The washed cells were suspended and maintained in RPMI media supplemented with 10% FBS, penicillin 100 U/mL, and streptomycin 100 g/mL at 37 °C and 5% CO_2_. Approximately 5 × 10^5^ cells were seeded into 24-well plates and allowed to equilibrate for 2 h. Pomalidomide or 3-MP, dissolved in 100% tissue culture grade dimethylsulfoxide (DMSO: Sigma Aldrich, St Louis, MO, USA), was then added to the wells. The final concentrations of drugs were 0.0, 0.6, 1.0, 10, 30, and 60 µM (*n* = 3–4 for each group). At 1 h after initiation of treatment, the cells were challenged with lipopolysaccharide (LPS, Sigma Aldrich, E.Coli 055:B5, Cat no. L4005-100MG, Lot no. 0000218759, Endotoxin Unit: 500,000 EU/mg, at a concentration of 10 ng/mL), and the media was collected after a further 24 h for evaluation of lactate dehydrogenase (LDH) activity as a marker of drug-induced cellular toxicity and concentrations of nitrite (In Vitro Toxicology Assay Kit, Lactic Dehydrogenase based, Promega, Cat no. G7891, Madison, WI, USA) (Griess Reagent System, Promega, Cat no. G2930) and TNF-α (human TNF-α ELISA MAX™ Deluxe Set Cat no. 430204, BioLegend, San Diego, CA, USA).

#### Target screening

To evaluate potential binding at classical neurological drug targets, 3-MP and 3,6’-DP were submitted to the National Institute on Mental Health (NIMH) Psychoactive Drug Screening Program (PDSP: Univ. N Carolina, Chapel Hill, NC, USA < https://pdspdb.unc.edu/pdspweb/ >) as powdered samples in the provided vials. The materials were then prepared at the PDSP in 10% DMSO, subjected to multiple assay screens, and processed as per the PDSP Assay Protocol Book (Version 3, March 2018). Compounds that exceeded an initial 10 uM binding threshold were then delegated for secondary dose–response analysis. This screen comprised a 45-target comprehensive panel of common brain receptor and transporter drug targets [36].

#### Ex vivo* metabolism*

Ex vivo evaluations of drug plasma and liver microsomal time-dependent breakdown are routinely undertaken to initially characterize the metabolic stability of new pharmacological compounds and were performed by WuXi AppTec (Shanghai, China; Study No. 424847–20,211,214-MMS). Rat and human plasma and liver microsomes were purchased from Xenotech (Sprague Dawley rat, Catalog no. R1000; BioIVT, Westbury, NY) or Corning (mixed human, Catalog no. 452117; Sigma). Rat and human plasma was incubated at 37 °C on a shaker and spiked with 3-MP (100 μg/ml, *n* = 3). Thereafter, samples were time-dependently (0 to 180 min) removed, immediately frozen (− 80 °C), and subjected to LC–MS/MS analysis at a later time.

Liver microsomes were diluted to 0.56 mg/mL in 100 mM phosphate buffer and added to pre-warmed (37 °C) 96-well format “Incubation” plates. 3-MP, together with three comparator compounds (testosterone, diclofenac, and propafenone) whose metabolic stabilities are known [37], was evaluated (all: final concentration 1 μM, prepared in DMSO (final concentration: 0.01% v:v)) in the presence and absence of the cofactor NADPH (120 μM prepared in MgCl_2_, Catalog no. BT04, Shenzhen, China). Samples were time-dependently collected (time: 0, 15, 30, 45, 60 min with NADPH and at 0 and 60 min without NADPH), and stop solution (acetonitrile containing internal standards) was added. The samples were centrifuged (3220 × *g*, 20 min, 4 °C); supernatant was collected, frozen (− 80 °C), and was later assayed for drug concentration by LC- MS/MS.

### C. Animal studies

All rats were housed at 25 °C in a 12 h light/12 h dark cycle and given free access to food and water. All efforts were made to minimize potential animal suffering and to decrease the number of animals used by integrating the outcome measures from our previous studies and use of a statistical power analysis. All described procedures were fully approved by the Institutional Animal Care and Use Committee of the Intramural Research Program, National Institute on Aging, NIH (protocols 331-TGB-2024 and 488-TGB-2022) for rat LPS studies and by the Taipei Medical University and the National Defense Medical Center (NDMC), Taipei, Taiwan (protocol IACUC-20–043) for stroke and related behavioral studies. All studies strictly followed NIH guidelines for research on rats. To avoid confounds consequent to the potential neuroprotective effects of estrogen, only male animals were used in this “first-in-animal” study of 3-MP.

#### Systemic and brain LPS anti-inflammatory studies

Adult male Fischer 344 rats (19 rats, approximately 150 g weight) were randomly assigned to five experimental groups (GraphPad, Boston, MA). They were administered, via the intraperitoneal (I.P.) route, nano-formulated 3-MP at a dose of 13.25 or 26.5 mg/kg (equimolar to a clinically translatable dose of 12.5 and 25 mg/kg thalidomide), vehicle or pomalidomide (25 mg/kg). One hour later, animals were administered either LPS (1 mg/kg, E.Coli 055:B5, Sigma Aldrich, in normal saline, 0.5 mg/mL) or an alike volume of saline (without LPS) via the I.P. route. The LPS dose was selected from prior pilot studies to provide a substantial but sub-maximal rise in plasma proinflammatory cytokine levels, in line with our prior research [29, 38, 39]. The selected 3-MP and pomalidomide doses were chosen as they are equimolar to, or are less than, doses of thalidomide and analogues that have been verified to be well tolerated in our previous studies [29, 39, 40] and are of translational relevance to humans. Four hours after LPS administration, the animals were euthanized, and blood and brain tissues were collected and placed on wet ice. Plasma was quickly separated from blood by centrifugation (10,000 × *g*, 5 min, 4 °C) and together with the brain samples (cerebral cortex) was stored at − 80 °C. At a later time, cerebral cortex samples were sonicated in a TRIS-based lysis buffer (Mesoscale Discovery) with 3 × protease/phosphatase inhibitors (Halt™ Protease and Phosphatase Inhibitor Cocktail, Thermo Fisher Scientific, Waltham, MA, USA). Samples were then centrifuged (10,000 × *g*, 5 min, 4 °C), supernatant separated, and protein concentrations were quantified by bicinchoninic acid assay (BCA, Thermo Fisher Scientific). Rat plasma and cerebral cortex samples were analyzed by multi-proinflammatory cytokine ELISA (V-PLEX Proinflammatory Panel 2 Rat Kit, Mesoscale Discovery) following the manufacturer’s protocol to quantify TNF-α, IL-6, IL-10, and IL-13 levels.

Rats (Fischer 344 males, approx. 150 g weight) were administered either by the I.P. or by oral (P.O.) route 3-MP (13.25 or 26.5 mg/kg). The animals were euthanized at 5 h post-dosing, and plasma and a brain sample were taken, frozen to − 80 °C, and later assayed for 3-MP by LC–MS/MS.

LC–MS/MS analysis was performed with a Vanquish Flex UHPLC (Thermo Scientific, Waltham MA, USA) and an Orbitrap Exploris 240 mass spectrometer (Thermo Scientific). Samples were separated on a C18 UHPLC column (100 mm × 2.1 mm, 3 μm; Thermo Scientific). The mobile phase A for LC separation was 0.1% formic acid and the mobile phase B was 0.1% formic acid in methanol. The chromatography gradient was initiated at 95% A and 5% B at zero time. This was continued for 1 min and, thereafter, linearly changed to 5% A and 95% B by 9 min. This was then maintained for a further 3 min. The flow rate was 0.3 mL/min, and the injection volume was 2 μl. Mass spectra were acquired using the data-dependent Top 4 MS2 (ddMS2) method with a mass scan (100–1000 m/z) and 120,000 MS resolution.

#### In vivo stroke studies employing the middle cerebral artery occlusion procedure

Male Sprague Dawley rats, weighing 195–210 g, were obtained from BioLASCO Taiwan for ischemic stroke studies. The MCAo procedure was performed as described previously [41–43]. Briefly, the rats were anesthetized using I.P.-administered zoletil + xylazine (Rompun) (25 mg/kg + 2.332 mg/kg). The bilateral common carotids were then ligated with non-traumatic arterial clips. A craniotomy of approximately 2 × 2 mm^2^ was applied to the right squamosal bone. Cerebral ischemia was induced by ligation of the right MCA with a 10-O suture thereby inducing MCAo for 60 min. The ligature and clips were removed after 60-min ischemia to generate re-perfusional injury. Core body temperature was monitored with a thermistor probe and maintained at 37 °C with a heating pad throughout anesthesia. After recovery from the anesthesia, body temperature was maintained at 37 °C.

The rats were subsequently randomly (GraphPad) divided into three experimental groups: vehicle, low dose 3-MP, and high dose 3-MP. Following 60-min MCAo and 30-min reperfusion, rats received I.P. injections of vehicle or 3-MP. The high and low 3-MP doses were 21.17 mg/kg and 10.58 mg/kg, respectively. However, since the “low” and “high” 3-MP doses produced very similar effects on the 2,3,5-triphenyltetrazolium chloride (TTC) staining analysis and are less than a quarter of a log value different, 3-MP dose data were combined in Fig. 6 in relation to the MCAo study.

#### Assessment of the injured cerebral lesion area and neurological deficit scores

Infarct size was measured based on Shen et al.[42] at 48 h after MCAo and was performed by an observer blinded to the treatment groups. Brains were quickly removed and immersed in cold saline for 5 min. Coronal slices were dissected into 2-mm segments from the frontal tips. Sections were immersed in 2% TTC at 37 °C for 10 min and subsequently fixed in a 5% formaldehyde solution. The brain infarct volume was calculated as a percentage zone of the coronal section in the infarcted hemisphere [42].

#### Behavior

Lateral movements and turning of the body were assessed using the body asymmetry (elevated body swing) test at 48 h, as detailed by Borlongan et al. [44, 45], by an observer blinded to the treatment groups. Specifically, rats were lifted by their tails and were maintained at a distance of approximately 20 cm above an examination table. The frequency of initial turning of the upper body contralateral to the ischemic side was calculated in 20 subsequent trials. The maximum impairment in body swing in MCAo-challenged rats is 20 contralateral turns/20 trials, in which animals demonstrate asymmetric behavior after MCAo [44]. By comparison, an uninjured animal, in general, shows a value of 10 (i.e., an equal number of left and right turns).

#### Cytokine evaluation in MCAo-challenged rats

Plasma was collected at 48 h to measure the levels of pro- and anti-inflammatory cytokines across treatment groups, with TNF-α, IL-1β, IL-6, and IL-10 being the proteins of interest. A Milliplex MAP Rat Cytokine/Chemokine Kit was used, per the manufacturer’s protocol. Samples were centrifuged, diluted as necessary in relation to prior pilot studies to align with the assay standard curves, and added to an ELISA plate. The samples were then incubated and washed, and antibody solutions were added. Following another incubation and wash, the enhanced chemiluminescence (ECL) signal levels were measured. The protein levels were determined using the MSD Discovery Workbench software. Protein concentrations were expressed as pg/ml.

### E. Data and statistical analysis

All data is shown as mean ± standard error of the mean (SEM) values, and all graphs were plotted using Prism. Numbers of samples/animals evaluated across studies were determined by incorporating the outcome measures from our prior studies [24, 29, 30] and a power analysis [46]. Data were assessed for the presence of statistical outliers (ROUT), and any observed outliers were removed. Data, additionally, were evaluated for normality (Shapiro–Wilk test). If normally distributed, they were assessed using an ordinary one-way ANOVA, followed by a Dunnett’s multiple comparison (Dunnett’s *t* test), or a Brown–Forsythe and Welch’s ANOVA test followed by the Dunnett’s T3 multiple comparison test. For non-normally distributed data, a non-parametric Kruskal–Wallis test followed by a Dunn’s multiple comparison test was utilized.

## Results

### 3-MP nanosuspension is time-dependently stable

**Fig. 1:**
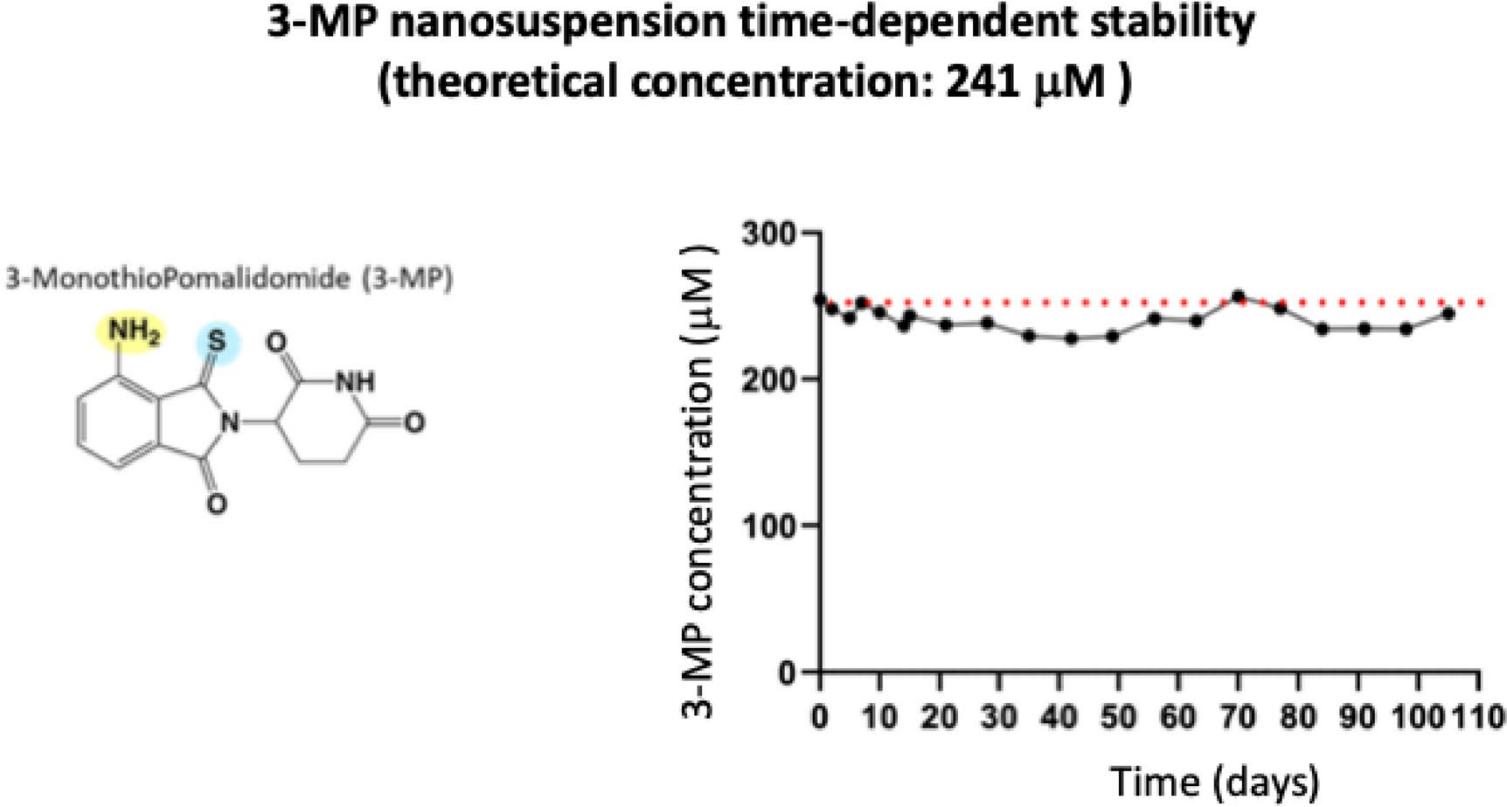
3-MP nanosuspension time-dependent stability. 3-MP was formulated in Tween-80/water (approx. 1:130 v/v), vortexed with yttrium-stabilized zirconia-silica beads, stored at 4 °C, sampled over a 98-day period and quantified by LC–MS

Consequent to the poor aqueous solubility of 3-MP and alike IMiDs, 3-MP was formulated into a nanosuspension to support its systemic administration in animal studies. To evaluate its chemical stability, 3-MP (241 μM, 4 °C) was time-dependently quantified over a 98-day period by LC–MS/MS (see Supplementary information). As illustrated in Fig. 1, 3-MP proved to be time-dependently stable over this duration.

### 3-MP mitigates LPS-induced inflammation in cultured human PBMCs

**Fig. 2:**
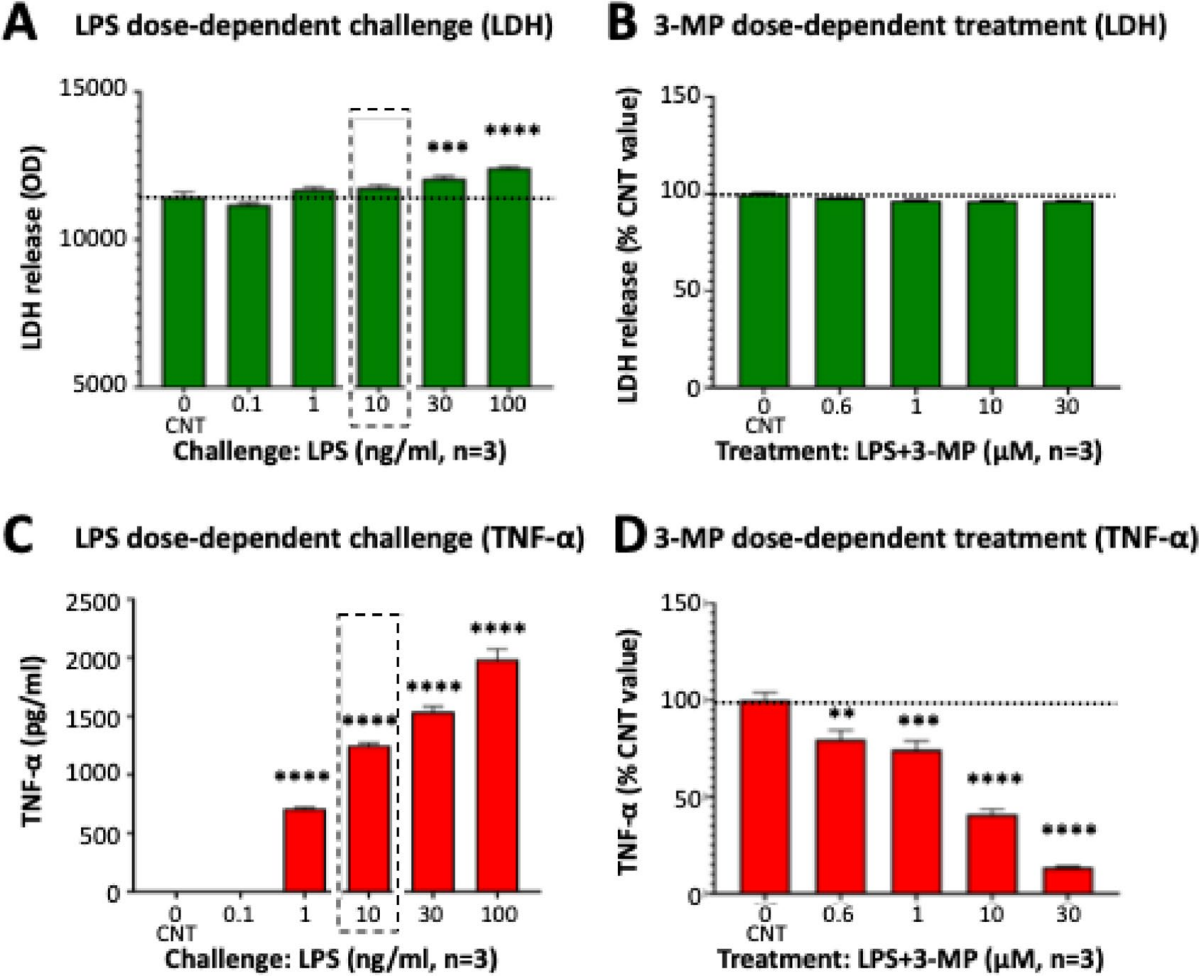
3-MP ameliorates LPS-induced inflammation in human PBMCs. Human PBMCs (5 × 10^5^ cells) dose-dependently challenged with LPS (0.1–100 ng/ml, 24 h) demonstrated a small elevation in LDH (**A**) and a substantial rise in TNF-α levels (**C**) in media versus vehicle-treated controls (CNT). Dose-dependent 3-MP treatment of PBMCs challenged with 10 ng/ml LPS resulted in significant declines in TNF-α (**D**) in the absence of LDH elevations (**B**). Data are presented as mean ± SEM (*n* = 3 culture wells per group). **p < 0.01; ***p < 0.001; and **** *p* < 0.0001 versus the control (CNT) group (one-way ANOVA + Dunnett’s *t* test)

As thalidomide has been demonstrated to selectively inhibit the production of TNF-α in LPS-challenged human PBMCs in vitro [47], the anti-inflammatory property of 3-MP was evaluated in an alike cellular model. As illustrated in Fig. 2 A and C, LPS challenge concentration-dependently elevated TNF-α levels and induced a small significant rise in LDH media levels following 24-h incubation with the higher LPS concentration. A well-tolerated (non-toxic) dose of 10 ng/ml LPS that induced a submaximal rise in TNF-α was selected to challenge human PBMCs in the presence of increasing concentrations of 3-MP (0 to 30 μM). 3-MP dose-dependently reduced TNF-α levels without elevating LDH media levels, as compared to the control (CNT: LPS alone) group (Fig. 2 B and D). In synopsis, 3-MP significantly mitigated LPS-induced inflammation in the absence of cellular toxicity, as evaluated by TNF-α and LDH media levels, respectively.

### 3-MP abates inflammation*in vivo*

**Fig. 3:**
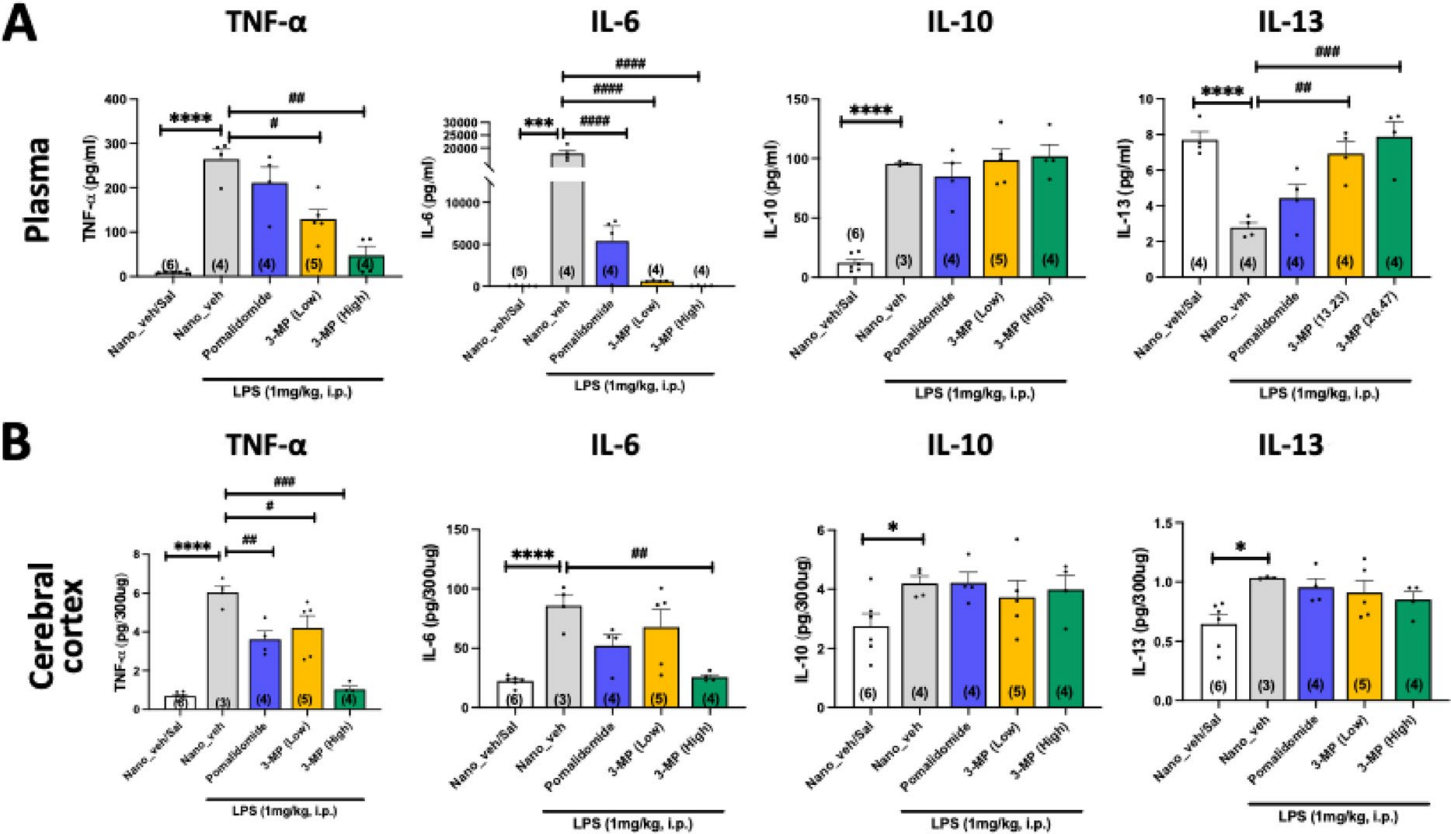
Effects of 3-MP on LPS-induced changes in proinflammatory and anti-inflammatory cytokines in plasma and cerebral cortex. Systemic administration of LPS (1 mg/kg, I.P.) to rats induced significant changes in pro- (TNF-α and IL-6) and anti-inflammatory cytokines (IL-10 and IL-13) at 4 h in both **A** plasma and **B** the brain (cerebral cortex). One-hour pre-treatment with 3-MP (low dose: 13.23 mg/kg; high dose: 26.47 mg/kg, I.P.) and pomalidomide (25 mg/kg, I.P.) significantly mitigated LPS-induced cytokine changes. *p < 0.05; ***p < 0.001; ****p < 0.0001 refers to the effects of LPS compared to the control value (Nano_veh + Sal: without LPS). #*p* < 0.05, ##*p* < 0.01, ###*p* < 0.001, ####*p* < 0.0001 refers to the effect of drug treatments vs. Nano_veh + LPS. Values are presented as mean ± S.E.M., of *n* observations (Nano_veh + Saline, *n* = 4–6; Nano_veh + LPS, *n* = 3–4; 3-MP (Low) + LPS, *n* = 4–5; 3-MP (High) + LPS, *n* = 4)

Since 3-MP dose-dependently mitigated LPS-induced inflammation in cell culture, as an initial in vivo approach, we evaluated the ability of 3-MP to reduce LPS-mediated increases in proinflammatory cytokines in rats. In line with prior studies from our group [27, 29, 39], systemic LPS administration (1 mg/kg, I.P.) provoked a substantial and statistically significant elevation in TNF-α and IL-6 plasma and brain levels, measured as representative proinflammatory cytokines, at 4 h. Changes in IL-10 and IL-13 were quantified as representative anti-inflammatory cytokines. The selection of a 4-h LPS challenge time was previously demonstrated to provide an approximate steady state for plasma TNF-α generation in response to LPS [39]. Both evaluated doses of 3-MP (low: 13.25 mg/kg; high: 26.5 mg/kg, I.P.) reduced LPS-induced rises in plasma TNF-α and IL-6 (*p* < 0.05 to < 0.0001), with the higher 3-MP dose demonstrating the greatest efficacy versus both the low 3-MP dose as well as an equimolar high dose of pomalidomide (Fig. 3A). An alike result was evident in the brain, albeit the 3-MP low dose and pomalidomide-mediated declines in IL-6 brain levels failed to achieve statistical significance (Fig. 3B).

In relation to anti-inflammatory cytokines, IL-10 elevation induced by LPS was, notably, not lessened by high- or low-dose 3-MP or pomalidomide in either plasma or brain. On the other hand, the LPS-mediated reduction in plasma IL-13 was abated by 3-MP (both doses: *p* < 0.01 to 0.001), and LPS-mediated elevations in brain IL-13 were maintained in animals dosed with 3-MP (both doses) and pomalidomide (Fig. 3 A and B).

### Drug metabolism/pharmacokinetic studies

To evaluate the metabolism of 3-MP, ex vivo plasma and liver homogenate studies were performed across species (rat and human), and in vivo pharmacokinetic studies were performed in rats following intravenous (I.V.) and oral (P.O.) administration of 3-MP (5 mg/kg) to time-dependently follow its disappearance from the plasma compartment.

**Fig. 4:**
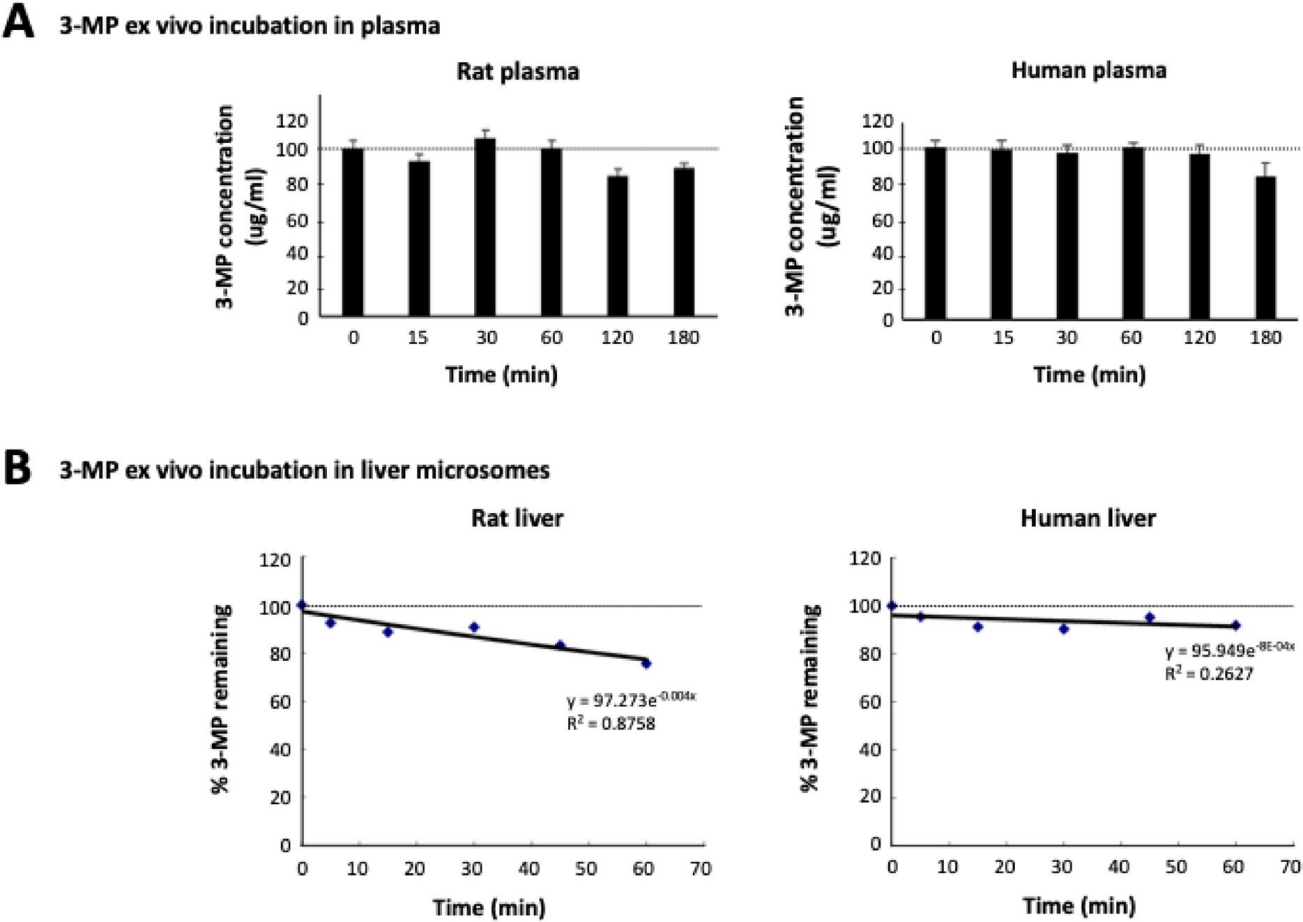
Ex vivo metabolic profile of 3-MP in rat and human plasma and liver microsomes. **A** Plasma incubation, and **B** liver incubation of 3-MP in the presence of the cofactor NADPH

As illustrated in Fig. 4A, 3-MP proved to be relatively stable when incubated in rat or human plasma and demonstrated minimal breakdown over the study duration (180 min). Similarly, in rat and human liver microsomes in the absence of NADPH, 3-MP demonstrated time-dependent chemical stability (Table 2. See: “Remaining (NCF = 60 min)”). To evaluate the involvement of phase-1 metabolism processes, NADPH was added as a cofactor to the liver microsome incubations. As evident in Fig. 4B, 3-MP levels were mildly reduced following 60-min incubation in activated rat microsomes, whereas levels remained relatively stable in human-derived microsomes over the same incubation period (by 24.3% and 8.2%, respectively). Calculated metabolic parameters are shown in Table 1, alongside three known comparator drugs whose metabolic profiles are well described in the scientific literature [48]. The metabolic disappearances of all three comparators align with literature values [48].

**Table 1:** Calculated metabolic parameters of 3-MP and comparator drugs following incubation in activated liver microsomes from rat (top) and human (bottom) (shown in Fig. 4B)

Compound	*R*^2^	*T*_1/2_	Cl_int(mic)_ (μl/min/mg)	Cl_int(liver)_ (ml/min/kg)	Remaining (*T* = 60 min)	Remaining (NCF = 60 min)
Rat						
3-MP	0.8758	> 145	< 9.6	< 17.3	75.7%	116.4%
Testosterone	1.0000*	0.6	2170.5	3906.8	0.1%	103.7%
Diclofenac	0.9994	11.0	126.3	227.4	2.2%	107.9%
Propafenone	0.9985	4.0	344.4	620.0	0.1%	107.9%
Human						
3-MP	0.2627	> 145	< 9.6	< 8.6	91.8%	115.3%
Testosterone	0.9827	21.1	65.6	59.0	13.2%	97.1%
Diclofenac	0.9979	4.3	324.8	292.3	0.0%	101.0%
Propafenone	0.9386	10.5	131.6	118.4	1.5%	108.7%

*R*^2^ (the correlation coefficient of the linear regression for the determination of kinetic data) was calculated from time 0- and 5-min values for testosterone in rat microsomes consequent to its rapid metabolism. All values (0 to 60 min) were used in *R*^2^ determinations for all other drugs. Specifically, no NADPH was added to NCF samples (replaced by buffer) during the 60-min incubation

*Remaining* % drug remaining at designated time, *NCF* no co-factor, *T*_*1/2*_ half-life, *Cl*_*int(mic)*_ intrinsic clearance, *Cl*_*int(mic)*_ 0.693/T_1/2_/mg microsome protein per mL, *Cl*_*int(liver)*_ Cl_int(mic)_ * mg microsomal protein/g liver weight * g liver weight/kg body weight

### Pharmacokinetics of 3-MP in the rat

**Fig. 5:**
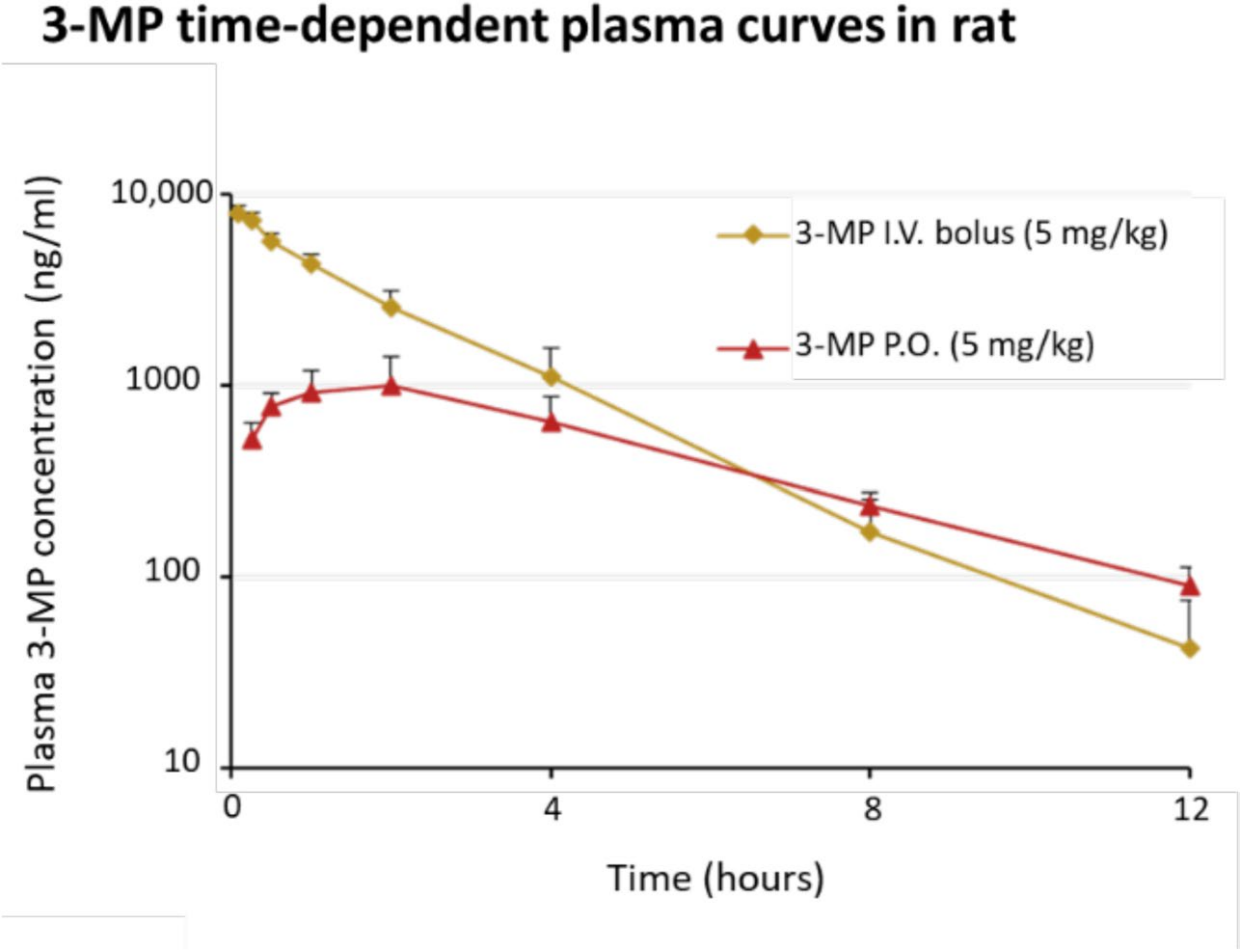
3-MP pharmacokinetics. 3-MP was administered to rats via the intravenous (I.V.) and oral (P.O.) routes, and time-dependent plasma levels were obtained and subjected to LC–MS/MS analysis (*n* = 3/group). Pharmacokinetic profile of 3-MP following a 5 mg/kg dose by either the I.V. or P.O route. Time-dependent plasma concentrations of 3-MP (*n* = 3/group)

As shown in Fig. 5, 3-MP disappears in a single compartmental manner from plasma following an I.V. bolus administration to rats with a *T*_1/2_ of 1.56 h. In contrast, following oral gavage of the same dose, 3-MP was absorbed and reached a peak concentration in plasma at 100 min and, thereafter, disappeared mono-phasically with a *T*_1/2_ of 3.21 h. Calculated pharmacokinetic parameters are shown in Table 2. Of note, the gastrointestinal bioavailability of 3-MP was 38.5%

**Table 2:** Calculated pharmacokinetic measures of 3-MP disappearance from rat plasma following I.V. versus P.O. dosing (*n* = 3/group)

Dose route	I.V. bolus		P.O	
3-MP dose (mg/kg)	5		5	
PK parameter	Mean	SEM	Mean	SEM
*C*_0_ or *C*_max_ (ng/ml)	8152	671	1024	257
*T*_max_ (h)	-	-	1.67	0.408
*T*_1/2_ (h)	1.56	0.207	3.21	0.653
Vd_ss_ (L/kg)	0.693	0.037	-	-
Cl (ml/min/kg)	5.65	0.884	-	-
AUC_0-last_ (ng h/ml)	15,128	2230	5559	996
AUC_0-inf_ (ng h/ml)	15,232	2292	5862	851
Bioavailability (%)	-	-	38.5	-

### 3-MP brain entry

**Fig. 6:**
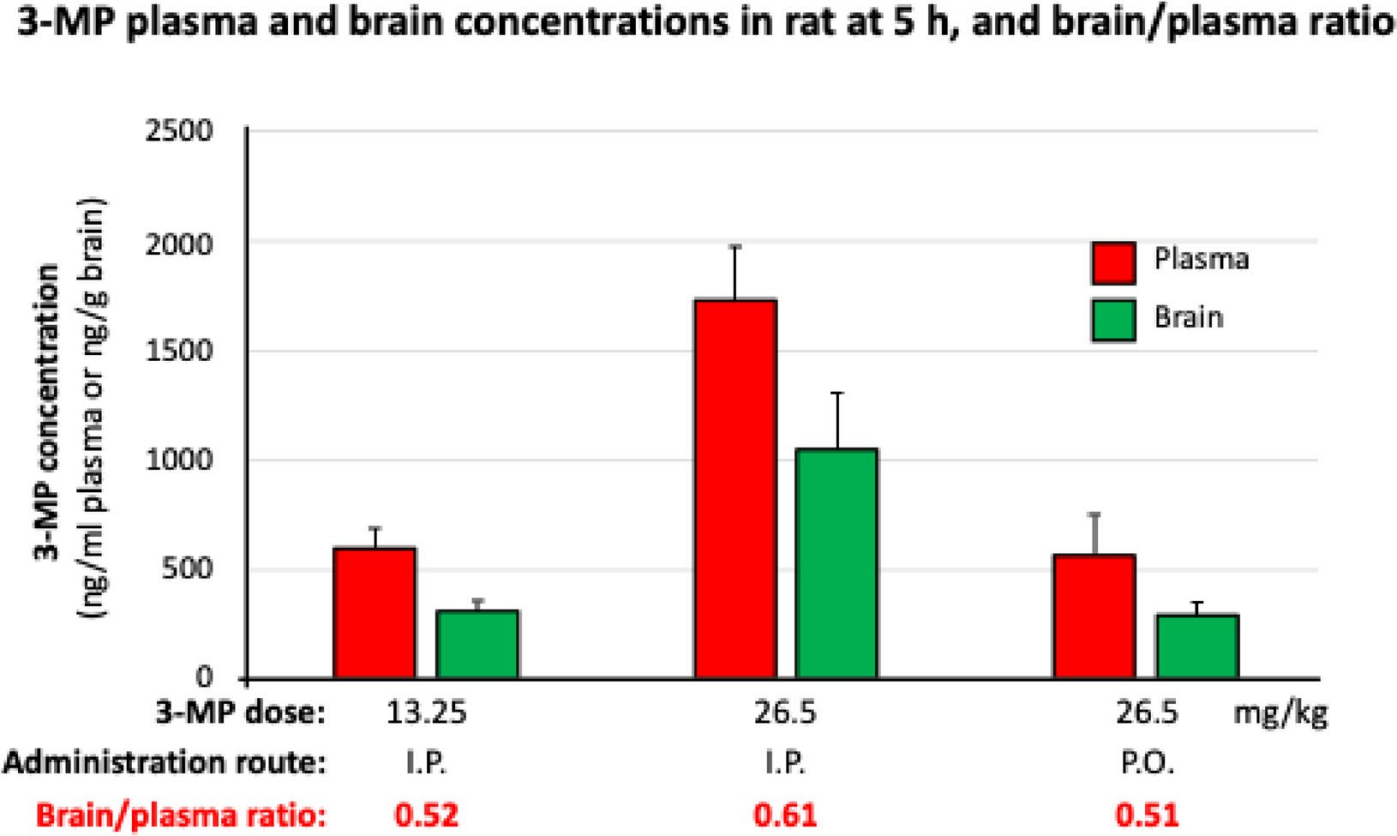
3-MP enters the brain following oral and intraperitoneal dosing. 3-MP was administered by the intraperitoneal (I.P.) and oral (P.O.) routes, and blood and brain samples were obtained at 5 h and subjected to LC–MS/MS evaluation (*n* = 4/group)

In a separate cohort of rats, 3-MP was administered either by the intraperitoneal (I.P.) or oral (P.O.) route at a dose of either 13.25 or 26.50 mg/kg. The animals were euthanized at 5 h post-dosing, and plasma and brain samples were analyzed for 3-MP by LC–MS/MS. As illustrated in Fig. 6, 3-MP readily entered the brain and achieved concentrations of 51 to 61% of concomitant plasma levels irrespective of the route of dosing. Notably, an oral dose of 26.5 mg/kg 3-MP generated similar plasma and brain concentrations as an I.P. dose of 13.25 mg/kg.

### 3-MP mitigates MCAo-induced ischemic damage and accompanying inflammation

**Fig. 7:**
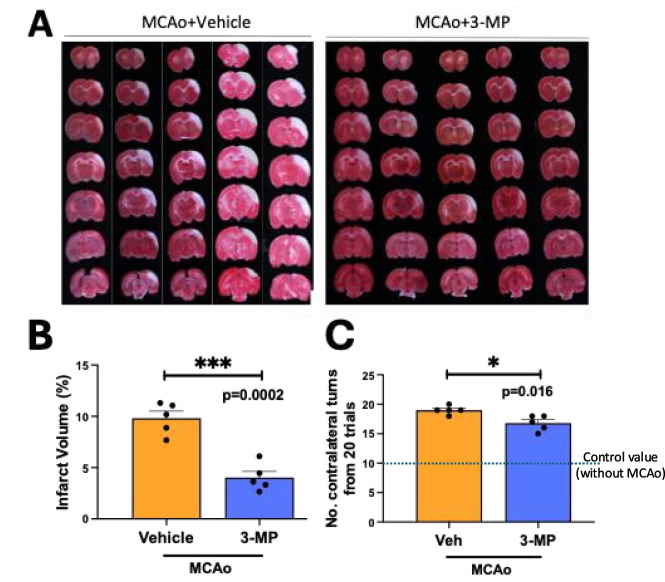
3-MP reduces brain infarction volume and behavioral impairment in rats. **A** TTC (2,3,5-triphenyltetrazolium chloride) staining of fresh brain tissue. **B** Quantification of brain infarct volume. **C** Resulting behavioral impairment (no. of contralateral turns from 20 trials). *p < 0.05; ***p < 0.001 vs vehicle treatment (*n* = 5/group)

Quantitation of brain infraction volume showed a marked reduction in the 3-MP group (Fig. 7 A and B). Specifically, the MCAo + 3-MP group had an infarct volume of 4.32 ± 1.33% versus the MCAo + vehicle group of 9.82 ± 1.53%, representing a 56% reduction. Body asymmetry was also reduced by drug treatment versus MCAo vehicle animals. The MCAo + 3-MP group exhibited 16.8 ± 1.3% contralateral turns, as compared to 19.00 ± 0.71% in the MCAo + vehicle group (Fig. 7C) from a total of 20 trials, with uncompromised control animals routinely generating 10 turns.

**Fig. 8:**
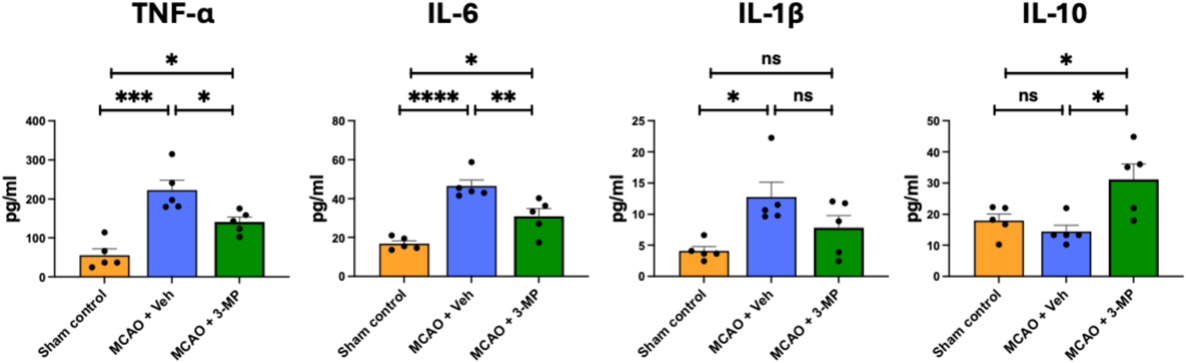
3-MP mitigates the systemic inflammatory response following cerebral ischemia. Forty-eight-hour systemic (plasma) levels of pro- and anti-inflammatory cytokines following 3-MP or vehicle administration to MCAo-challenged rats. *p < 0.05; **p < 0.01; ***p < 0.001, n.s. no statistical difference (one-way ANOVA + followed by a Dunnett’s multiple comparison (Dunnett’s *t* test)); *n* = 5/group)

Either 3-MP or vehicle was administered to animals challenged with MCAo at 60 min after occlusion and reperfusion. As illustrated in Fig. 8, plasma samples were obtained 48 h later, and TNF-α, IL-6, IL-1β, and IL-10 levels were quantified (Milliplex platform). In this regard, the acute phase of ischemic stroke leads to the generation and release of pro-inflammatory cytokines, which time-dependently propagate the neuroinflammatory response. Brain ischemia induced significant elevations in plasma levels of the pro-inflammatory TNF-α, IL-6, and IL-1β, which were significantly mitigated by 3-MP. Importantly, this was accompanied by a 3-MP-mediated rise in anti-inflammatory IL-10 levels.

### Activity against brain classical targets

To evaluate potential “off target” actions against classical neurological receptor and transporter drug targets that could potentially underpin beneficial or adverse drug actions of 3-MP use, screening in the radioligand binding PDSP was performed. A total of 45 targets (42 receptor and 3 transporter subtypes) were assessed (Table 3). An initial screen was undertaken at a concentration of 10 μM, and quantification of binding inhibition of the natural radioligand to each target was determined. With the exception of the Alpha1A, Sigma 2, and H4 receptors, radioligand displacement was negligible for all other targets. A secondary, concentration-dependent evaluation of 3-MP (0.01 nM to10μM) action at these three receptors resulted in an approx. 50% inhibition of radioligand receptor binding at the highest 3-MP (10 μM) concentration evaluated and negligible action at the other two. These data are indicative of 3-MP inactivity across the evaluated neurological drug targets.

**Table 3:** 3-MP concentration required to induce 50% inhibition of PDSP radioligand binding

Receptors				
Serotonin	Adrenergic	Dopamine	Histamine	Sigma
5-HT1A > 10 μM	Alpha1A > 10 μM	D1 > 10 μM	H1 > 10 μM	Sigma 1 > 10 μM
5-HT1B > 10 μM	Alpha1B > 10 μM	D2 > 10 μM	H2 > 10 μM	Sigma 2 > 10 μM
5-HT1D > 10 μM	Alpha1D > 10 μM	D3 > 10 μM	H3 > 10 μM	
5-HT1E > 10 μM	Alpha2A > 10 μM	D4 > 10 μM	H4 > 10 μM	
5-HT2A > 10 μM	Alpha2B > 10 μM	D5 > 10 μM		
5-HT2B > 10 μM	Alpha2C > 10 μM		Muscarinic	
5-HT2C > 10 μM	Beta1 > 10 μM	Opioid	M1 > 10 μM	
5-HT3 > 10 μM	Beta2 > 10 μM	δ-opioid > 10 μM	M2 > 10 μM	
5-HT5A > 10 μM	Beta3 > 10 μM	κ-opioid > 10 μM	M3 > 10 μM	
5-HT6 > 10 μM		μ-opioid > 10 μM	M4 > 10 μM	Transporters
5-HT7A > 10 μM	Benzodiazepine		M5 > 10 μM	DAT > 10 μM
	BZP > 10 μM	GABA_A_		NET > 10 μM
	PBR > 10 μM	GABA_A_ > 10 μM		SERT > 10 μM

## Discussion

Time-dependently following cerebral ischemia, a range of deleterious processes can occur that include blood–brain barrier damage, oxidative stress, inflammation, neuronal death, and resulting neurological impairments [7–15]. It has become increasingly recognized that an increased understanding of the intricacies of post-stroke inflammation is necessary to aid the development of targeted therapies to modulate the immune response and promote neuroprotection and repair in stroke survivors. Our previous research demonstrated that the IMiD, pomalidomide [24], and potentially less toxic analogues, such as 3,6’-DP and 1,6’-DP [30], have neuroprotective effects in the widely used preclinical MCAo model of cerebral ischemia. Notably, such novel IMiD analogues provide anti-inflammatory action and efficacy across preclinical models of neurodegenerative disorders that possess a prominent neuroinflammatory component, such as also occurs in AD and traumatic brain injury [26, 27, 29, 31, 32]. However, although efficacious when administered once daily, and readily entering the brain (brain/plasma concentration ratio 0.8 [26]), the lead compound (3,6’-DP) amongst these novel pomalidomide analogues disappears rapidly from plasma following administration to rodents. Pharmacological efficacy extending beyond the in vivo existence of a drug is a phenomenon often associated with the presence of one or more active metabolites. As a consequence, the current study evaluated the pharmacokinetic and biological action of 3-MP, a predicted 3,6’-DP metabolite, as a new IMiD to alleviate neuroinflammation. Our results demonstrate that 3-MP has the potential to mitigate a classical inflammatory response induced by LPS in cultured human PBMCs, and that this action translates in vivo to rats challenged with LPS — both systemically and within the brain. Furthermore, 3-MP reduced brain infarction volume, evaluated by TTC staining, mitigated motor impairment, and reduced cytokine-related inflammation in a classical MCAo model of ischemic and reperfusion injury when administered at a dose of clinical equivalence in relation to thalidomide [39, 40]. Additionally, 3-MP proved to be relatively stable when incubated in rat or human plasma and demonstrated low breakdown in activated liver homogenates. This translated into a predictable pharmacokinetic profile following intravenous and oral administration to rats, suggestive of drug-like properties. In synopsis, our research highlights 3-MP as a lead compound to mitigate excessive inflammation and associated damage that can ensue following cerebral ischemia.

The most prominent drugs of the IMiD class are thalidomide, lenalidomide, and pomalidomide that are clinically approved and routinely used in the treatment of multiple myeloma. Their antiproliferative action is mediated via binding and modifying the action of human cereblon [49–51], a neosubstrate recruiter/receptor within the cullin-RING ligase-4 E3 ligase complex [50, 51]. This is one of multiple E3 ligases that form the ubiquitin–proteasome system (UPS), which has a critical role in degrading intracellular proteins; these include the transcription factors Ikaros and Aiolos of relevance in IMiD multiple myeloma treatment [49–52]. X-ray crystallographic studies have defined a shallow pocket, termed the thalidomide binding domain, on the surface of human cereblon with which IMiDs possessing a glutarimide ring interact [19, 51, 53–56]. This pocket centers around three tryptophan residues with a phenylalanine side-chain forming the base of the binding pocket [53–56]. Following IMiD glutarimide ring binding within the pocket, the molecule orientates the variable phthalimide/isoindolinone portion of the IMiD to protrude beyond the surface of cereblon [50–56] to, thereby, create a “hot-spot” to support key protein/protein interactions that ultimately result in their ubiquitination and degradation. Minor structural modifications in IMiDs can modify both their human cereblon binding and support of subsequent protein ubiquitin tagging and degradation [50, 51, 56].

Whereas thalidomide and close analogues can bind rodent cereblon [18, 19], degradation of rodent transcription factors, such as Ikaros and Aiolos, does not occur, and murine multiple myeloma cells are insensitive to IMID treatment [57]. In this regard, a single amino acid within the cereblon-IMiD binding domain results in the resistance of rodent cereblon in relation to the degradative E3 ligase actions associated with IMiDs [58]. Hence, in relation to IMiD anti-cancer actions, rodent processes cannot be readily generalized to humans [59].

IMiDs additionally reduce the generation of proinflammatory cytokines via cereblon-dependent and independent actions [60, 61]. On this basis, the new analogues 3,6’-DP and the focus of the current study, 3-MP, were discovered using a phenotypic (anti-inflammatory) drug discovery approach [28]. Whereas both of these new IMiDs possess a glutarimide-like ring, other IMiD-based compounds that we have generated, such as *N*-adamantyl phthalimidine (NAP) and tetrafluorobornylphthalimide (TFBP), possess a larger cage-like ring that precludes their binding to cereblon and yet retains anti-inflammatory actions [25–27, 29–32, 62]. In further support of cereblon-independent anti-inflammatory actions, thalidomide, lenalidomide, and pomalidomide have been shown to mitigate LPS-induced inflammation in both normal and cereblon knockout mice [61]. Likewise, anti-inflammatory actions are retained in a model of ulcerative colitis involving both normal and humanized cereblon mice [58]. In relation to anti-inflammatory mechanisms, IMiDs are widely considered to alter the stability of TNF-α mRNA [63] and have a mild inhibitory effect on NFκB and AP-1 (activating protein-1) activation that would contribute to the repression of TNF-α [64–67]. Furthermore, across both normal and cereblon knockout mice, IMiDs inhibit toll-like receptor (TLR)4-induced cytokine production via suppression of the TRIF/IRF3 pathway [60, 61]. This is of particular relevance to LPS which mediates its proinflammatory actions, in large part, via TLR4 signaling, the E3 ubiquitin ligase TNF receptor-associated factor 6 (TRAF6) and the transforming growth factor β–activated kinase 1 (TAK1) pathways [68]. Furthermore, ischemia reperfusion injury is considered to be mediated, in large part, via TLR4 as well as TLR2, with knockout abating injury and over-expression amplifying it [69].

It has additionally, been demonstrated that cereblon has a role in inflammation, including that triggered by LPS. Specifically, Min et al. [70] demonstrated that sensitivity to LPS challenge was significantly elevated in cereblon-knockout mice and associated with increased TNF-α and IL-6 levels, indicating a negative regulatory role of cereblon on TLR4 signaling. Specifically, cereblon appears to bind to the ubiquitination domain of TRAF6 and, thereby, attenuate TRAF6 and TAK1-binding protein 2 (TAB2) ubiquitination; this can ultimately repress NF-κB activation [70]. This appears to be a nonenzymatic function of cereblon. Another mechanism is its role in reducing the formation of protein aggregates, which often trigger inflammation, in neurodegenerative pathological disorders [71].

Cytokines play a pivotal role in the inflammatory response following an ischemic stroke with TNF-α, IL-1β, IL-6, and IL-10 being particularly significant [9–15]. TNF-α, predominantly released by activated microglia and infiltrating immune cells, acts as a potent initial pro-inflammatory mediator, contributing to blood–brain barrier disruption, leukocyte infiltration, and neuronal injury. It has been observed that IMiDs, such as pomalidomide, can modulate this inflammatory response [25, 32]. In this regard, activation of macrophages results in multiple log-fold increases in TNF-α biosynthesis with only a modest threefold increase in transcription [72]. Hence, TNF-α expression is chiefly regulated at the level of mRNA stability and translation [73]. Such post-transcriptional management of TNF-α expression is, in large part, regulated via an adenine-uridine-rich element (ARE) present within its 3′-untranslated region (3′-UTR) [72, 73]. By dampening TNF-α generation and release, IMiDs can potentially reduce the overall inflammatory cascade, thereby minimizing the extent of neuronal damage and improving outcomes in ischemic stroke [23, 25, 30, 32]. A reduction in TNF-α levels leads to decreased activation of NF-κB, a key factor that promotes inflammation. Modulation of TNF-α by IMiDs appears to balance the immune response, enhancing the activity of T-cells and natural killer (NK) cells, thus further supporting neuroprotection and tissue repair [74].

Similarly, IL-1β leads to neuroinflammation, causing neuronal damage and secondary injury mechanisms [75]. In contrast, IL-10 has anti-inflammatory effects to inhibit proinflammatory cytokine production, to suppress microglial activation, and to promote tissue repair and neurogenesis [76]. IL-6 has dual effects, exacerbating infarct size and outcomes during the acute phase while promoting tissue repair, neurogenesis, and angiogenesis during the subacute and chronic phases of stroke recovery [77]. Hence, maintaining a balance between proinflammatory and anti-inflammatory cytokines is crucial to optimize clinical outcomes in ischemic stroke. Higher levels of proinflammatory cytokines and lower levels of anti-inflammatory IL-10 are associated with larger infarct sizes and a worse prognosis [78]. Our previous studies showed that 3,6’-DP and 1,6’-DP significantly reduce IL-1β and TNF-α levels in animals with MCAo [30]. Notably, IL-10 levels were increased substantially in animals treated with MCAo + 3,6’-DP [30]. This current study, likewise, found that 3-MP’s neuroprotective effects are achieved acutely by decreasing TNF-α and IL-6 levels while increasing IL-10 to restore balance in the post-ischemic inflammatory response; this action was largely replicated in our LPS cellular and in vivo studies. This action likely involves central and peripheral immune mechanisms to address the complex interplay between systemic inflammation and central nervous system pathology in stroke [79].

Pharmacokinetic studies demonstrate that 3-MP has drug-like properties and maintains a stable profile in plasma and activated liver microsomes, with minimal breakdown over the duration of our study. Furthermore, after in vivo administration, 3-MP is rapidly absorbed, with an estimated oral bioavailability of approximately 38.5% in rats. In contrast, the reported bioavailability of pomalidomide in rat is 13% and in nonhuman primate (cynomolgus monkey) is 15% [80]. Notably, unlike traditional anti-inflammatory drugs such as ibuprofen and aspirin that have been investigated for their potential neuroprotective effects in stroke models but have been limited by adverse effects and their limited ability to cross the blood–brain barrier [81], the brain/plasma concentration ratio of 3-MP (0.51 to 0.61) is similar to that of Pom (0.71 [25], 0.39–0.41 [80, 82]). This indicates 3-MP’s ability to readily penetrate the blood–brain barrier and have central nervous system action [25, 32]. An initial evaluation of PDSP receptor binding indicates minimal interaction of 3-MP with the assessed brain targets, and thus does not reveal concerns with regard to potential adverse neurological actions. However, longer-term focused in vivo studies are needed to evaluate this.

Our study has several limitations, including the use of a single animal species (rat) and the relatively short duration of experimental studies (up to 48 h). Additionally, our investigation, as the first in vivo studies, focused on males, necessitating future research to include females to elucidate gender-specific effects. Both preclinical and clinical studies have revealed male/female differences in ischemic stroke incidence and outcome [83] and showed that estrogen can attenuate cerebral damage caused by an ischemic stroke — possessing potential immunomodulatory actions [84–86]. Multiple animal and human studies have demonstrated age-associated microglial and glial cell functional impairments that underpin the development of a chronic neuroinflammatory state with immunosenescence, which can lead to elevated levels of proinflammatory cytokines and disproportionate innate and adaptive responses [87–89]. In this light, evaluation of the actions of 3-MP in aged animal models would be valuable. Based on the promising data in the current studies, addressing these limitations in future studies will provide a more comprehensive understanding of 3-MP’s therapeutic potential. Future research should focus on the long-term effects of 3-MP treatment across gender and age, identifying biomarkers for treatment response. There should also be more detailed pharmacodynamic studies across neurodegenerative diseases in line with neurological drug development [33]. In synopsis, 3-MP shows promise as a therapeutic agent for mitigating neuroinflammation and reducing neuronal damage in ischemic stroke. Further preclinical investigations are necessary to validate our current findings and explore the translational potential of 3-MP to mitigate ischemic stroke and other conditions with a prevalent neuroinflammatory component.

## Supplementary Information

Below is the link to the electronic supplementary material.

Supplementary file1 (DOCX 2035 KB)

## References

[1] Collins TR. Neurologic diseases found to be the largest cause of disability worldwide. Neurology Today. 2017;17(22):1–32.

[2] Doyle KP, Simon RP, Stenzel-Poore MP. Mechanisms of ischemic brain damage. Neuropharmacology. 2008;55(3):310–8. 18308346 10.1016/j.neuropharm.2008.01.005 PMC2603601

[3] Kelly PJ, Lemmens R, Tsivgoulis G. Inflammation and stroke risk: a new target for prevention. Stroke. 2021;52(8):2697–706. 34162215 10.1161/STROKEAHA.121.034388

[4] Petrovic-Djergovic D, Goonewardena SN, Pinsky DJ. Inflammatory disequilibrium in stroke. Circ Res. 2016;119(1):142–58. 27340273 10.1161/CIRCRESAHA.116.308022 PMC5138050

[5] Yousufuddin M, Young N. Aging and ischemic stroke. Aging (Albany NY). 2019;11(9):2542–4. 10.18632/aging.101931. 31043575 10.18632/aging.101931 PMC6535078

[6] Singh V, Cheng R. Neurovascular physiology and neurocritical care. Handb Clin Neurol. 2021;176:71–80. 10.1016/B978-0-444-64034-5.00014-6. 33272411 10.1016/B978-0-444-64034-5.00014-6

[7] Stuckey SM, Ong LK, Collins-Praino LE, Turner RJ. Neuroinflammation as a key driver of secondary neurodegeneration following stroke? Int J Mol Sci. 2021;22(23):13101. 10.3390/ijms222313101. 34884906 10.3390/ijms222313101 PMC8658328

[8] Yang C, Hawkins KE, Doré S, Candelario-Jalil E. Neuroinflammatory mechanisms of blood-brain barrier damage in ischemic stroke. Am J Physiol Cell Physiol. 2019;316(2):C135–53. 10.1152/ajpcell.00136.2018. 30379577 10.1152/ajpcell.00136.2018 PMC6397344

[9] Jayaraj RL, Azimullah S, Beiram R, Jalal FY, Rosenberg GA. Neuroinflammation: friend and foe for ischemic stroke. J Neuroinflammation. 2019;16(1):142. 10.1186/s12974-019-1516-2. 31291966 10.1186/s12974-019-1516-2 PMC6617684

[10] Xu S, Lu J, Shao A, Zhang JH, Zhang J. Glial cells: role of the immune response in ischemic strokE. Front Immunol. 2020;11:294. 10.3389/fimmu.2020.00294. 32174916 10.3389/fimmu.2020.00294 PMC7055422

[11] Maida CD, Norrito RL, Daidone M, Tuttolomondo A, Pinto A. Neuroinflammatory mechanisms in ischemic stroke: focus on cardioembolic stroke, background, and therapeutic approaches. Int J Mol Sci. 2020;21(18):6454. 10.3390/ijms21186454. 32899616 10.3390/ijms21186454 PMC7555650

[12] Fassbender K, Rossol S, Kammer T, Daffertshofer M, Wirth S, Dollman M, Hennerici M. Proinflammatory cytokines in serum of patients with acute cerebral ischemia: kinetics of secretion and relation to the extent of brain damage and outcome of disease. J Neurol Sci. 1994;122(2):35–9. 10.1016/0022-510x(94)90289-5. 10.1016/0022-510x(94)90289-5 8021695

[13] Kumari S, Dhapola R, Sharma P, Nagar P, Medhi B, HariKrishnaReddy D. The impact of cytokines in neuroinflammation-mediated stroke. Cytokine Growth Factor Rev. 2024;78:105–19. 10.1016/j.cytogfr.2024.06.002. 39004599 10.1016/j.cytogfr.2024.06.002

[14] Drieu A, Levard D, Vivien D, Rubio M. Anti-inflammatory treatments for stroke: from bench to bedside. Ther Adv Neurol Disord. 2018;11:1756286418789854. 10.1177/1756286418789854. 30083232 10.1177/1756286418789854 PMC6066814

[15] Vila N, Castillo J, Dávalos A, Esteve A, Planas AM, Chamorro A. Levels of anti-inflammatory cytokines and neurological worsening in acute ischemic stroke. Stroke. 2003;34:671–5. 10.1161/01.STR.0000057976.53301.69. 12624290 10.1161/01.STR.0000057976.53301.69

[16] García-Poza P, de Abajo FJ, Gil MJ, Chacón A, Bryant V, García-Rodríguez LA. Risk of ischemic stroke associated with non-steroidal anti-inflammatory drugs and paracetamol: a population-based case-control study. J Thromb Haemost. 2015;13(5):708–18. 10.1111/jth.12855. 25611553 10.1111/jth.12855

[17] U.S. Food and Drug Administration. FDA Drug Safety Communication: FDA strengthens warning that non-aspirin nonsteroidal anti-inflammatory drugs (NSAIDs) can cause heart attacks or strokes. https://www.fda.gov/drugs/drug-safety-and-availability/fda-drug-safety-communication-fda-strengthens-warning-non-aspirin-nonsteroidal-anti-inflammatory. 7.9.2015 (accessed 11.25.2024)

[18] Raza S, Safyan RA, Lentzsch S. Immunomodulatory drugs (IMiDs) in multiple myeloma. Curr Cancer Drug Targets. 2017;17(9):846–57. 10.2174/1568009617666170214104426. 28201976 10.2174/1568009617666170214104426

[19] Chamberlain PP, Cathers BE. Cereblon modulators: low molecular weight inducers of protein degradation. Drug Discov Today Technol. 2019;31:9–34. 10.1016/j.ddtec.2019.02.004. 10.1016/j.ddtec.2019.02.004 31200856

[20] Sheskin J. Thalidomide in the treatment of lepra reactions. Clin Pharmacol Ther. 1965;6:303–6. 10.1002/cpt196563303. 14296027 10.1002/cpt196563303

[21] de Jesus SM, Santana RS, Leite SN. Comparative analysis of the use and control of thalidomide in Brazil and different countries: is it possible to say there is safety? Expert Opin Drug Saf. 2022;21(1):67–81. 10.1080/14740338.2021.1953467. 34232089 10.1080/14740338.2021.1953467

[22] Jiang Y, Zou Y, Wang H. Review of research progress on different modalities of Macrophage death in Mycobacterium leprae infection. Int Immunopharmacol. 2024;142(Pt B):113240. 10.1016/j.intimp.2024.113240. 39332094 10.1016/j.intimp.2024.113240

[23] Tsai YR, Chang CF, Lai JH, Wu JC, Chen YH, Kang SJ, Hoffer BJ, Tweedie D, Luo W, Greig NH, Chiang YH, Chen KY. Pomalidomide ameliorates H(2)O(2)-induced oxidative stress injury and cell death in rat primary cortical neuronal cultures by inducing anti-oxidative and anti-apoptosis effects. Int J Mol Sci. 2018;19:3252. 10.3390/ijms19103252. 30347766 10.3390/ijms19103252 PMC6213994

[24] Tsai YR, Tweedie D, Navas-Enamorado I, Scerba MT, Chang CF, Lai JH, Wu JC, Chen YH, Kang SJ, Hoffer BJ, de Cabo R, Greig NH, Chiang YH, Chen KY. Pomalidomide reduces ischemic brain injury in rodents. Cell Transplant. 2019;28(4):439–50. 10.1177/0963689719850078. 31094216 10.1177/0963689719850078 PMC6628558

[25] Jung YJ, Tweedie D, Scerba MT, Greigm NH. Neuroinflammation as a factor of neurodegenerative disease: thalidomide analogs as treatments. Front Cell Dev Biol. 2019;7:313. 10.3389/fcell.2019.00313. 31867326 10.3389/fcell.2019.00313 PMC6904283

[26] Lin CT, Lecca D, Yang LY, Luo W, Scerba MT, Tweedie D, Huang PS, Jung YJ, Kim DS, Yang CH, Hoffer BJ, Wang JY, Greig NH. 3,6’-dithiopomalidomide reduces neural loss, inflammation, behavioral deficits in brain injury and microglial activation. Elife. 2020;9:e54726. 10.7554/eLife.54726. 32589144 10.7554/eLife.54726 PMC7375814

[27] Hsueh SC, Luo W, Tweedie D, Kim DS, Kim YK, Hwang I, Gil JE, Han BS, Chiang YH, Selman W, Hoffer BJ, Greig NH. N-adamantyl phthalimidine: a new thalidomide-like drug that lacks cereblon binding and mitigates neuronal and synaptic loss, neuroinflammation, and behavioral deficits in traumatic brain injury and LPS challenge. ACS Pharmacol Transl Sci. 2021;4:980–1000. 10.1021/acsptsci.1c00042. 33860215 10.1021/acsptsci.1c00042 PMC8033775

[28] Vincent F, Nueda A, Lee J, Schenone M, Prunotto M, Mercola M. Phenotypic drug discovery: recent successes, lessons learned and new directions. Nat Rev Drug Discov. 2022;21(12):899–914. 10.1038/s41573-022-00472-w. 35637317 10.1038/s41573-022-00472-w PMC9708951

[29] Lecca D, Jung YJ, Scerba MT, Hwang I, Kim YK, Kim S, Modrow S, Tweedie D, Hsueh SC, Liu D, Luo W, Glotfelty E, Li Y, Wang JY, Luo Y, Hoffer BJ, Kim DS, McDevitt RA, Greig NH. Role of chronic neuroinflammation in neuroplasticity and cognitive function: a hypothesis. Alzheimers Dement. 2022;18:2327–40. 10.1002/alz.12610. 35234334 10.1002/alz.12610 PMC9437140

[30] Tsai YR, Kim DS, Hsueh SC, Chen KY, Wu JC, Wang JY, Tsou YS, Hwang I, Kim Y, Gil D, Jo EJ, Han BS, Tweedie D, Lecca D, Scerba MT, Selman WR, Hoffer BJ, Greig NH, Chiang YH. 3,6’- and 1,6’-dithiopomalidomide mitigate ischemic stroke in rats and blunt inflammation. Pharmaceutics. 2022;14(5):950. 10.3390/pharmaceutics14050950. 35631536 10.3390/pharmaceutics14050950 PMC9146426

[31] Huang PS, Tsai PY, Yang LY, Lecca D, Luo W, Kim DS, Hoffer BJ, Chiang YH, Greig NH, Wang JY. 3,6’-dithiopomalidomide ameliorates hippocampal neurodegeneration, microgliosis and astrogliosis and improves cognitive behaviors in rats with a moderate traumatic brain injury. Int J Mol Sci. 2021;22:8276. 10.3390/ijms22158276. 34361041 10.3390/ijms22158276 PMC8348060

[32] Kopp KO, Greer ME, Glotfelty EJ, Hsueh SC, Tweedie D, Kim DS, Reale M, Vargesson N, Greig NH. A new generation of IMiDs as treatments for neuroinflammatory and neurodegenerative disorders. Biomolecules. 2023;13(5):747. 10.3390/biom13050747. 37238617 10.3390/biom13050747 PMC10216254

[33] Mohs RC, Greig NH. Drug discovery and development: role of basic biological research. Alzheimers Dement (N Y). 2017;3(4):651–7. 10.1016/j.trci.2017.10.005. 29255791 10.1016/j.trci.2017.10.005 PMC5725284

[34] Brown GC. Neuronal loss after stroke due to microglial phagocytosis of stressed neurons. Int J Mol Sci. 2021;22(24):13442. 10.3390/ijms222413442. 34948237 10.3390/ijms222413442 PMC8707068

[35] Cardia CM, Palmas FM, Casula L, Pisanu A, Marceddu S, Valenti D, Sinico C, Pini E, Scerba MT, Tweedie D, Greig NH, Carta AR, Lai F. Nanocrystals as an effective strategy to improve pomalidomide bioavailability in rodent. Int J Pharm. 2022;625:122079. 10.1016/j.ijpharm.2022.122079. 35932932 10.1016/j.ijpharm.2022.122079

[36] Besnard J, Ruda GF, Setola V, Abecassis K, Rodriguiz RM, Huang XP, Norval S, Sassano MF, Shin AI, Webster LA, Simeons FR, Stojanovski L, Prat A, Seidah NG, Constam DB, Bickerton GR, Read KD, Wetsel WC, Gilbert IH, Roth BL, Hopkins AL. Automated design of ligands to polypharmacological profiles. Nature. 2012;492(7428):215–20. 10.1038/nature11691.10.1038/nature11691. 23235874 10.1038/nature11691 PMC3653568

[37] Asadi F, Gunawardana SC, Dolle RE, Piston DW. An orally available compound suppresses glucagon hypersecretion and normalizes hyperglycemia in type 1 diabetes. JCI Insight. 2024;9(2):e172626. 10.1172/jci.insight.172626. 38258903 10.1172/jci.insight.172626 PMC10906223

[38] Tweedie D, Luo W, Short RG, Brossi A, Holloway HW, Li Y, Yu QS, Greig NH. A cellular model of inflammation for identifying TNF-alpha synthesis inhibitors. J Neurosci Methods. 2009;183(2):182–7. 10.1016/j.jneumeth.2009.06.034. 19583982 10.1016/j.jneumeth.2009.06.034 PMC2756970

[39] Tweedie D, Ferguson RA, Fishman K, Frankola KA, Van Praag H, Holloway HW, Luo W, Li Y, Caracciolo L, Russo I, Barlati S, Ray B, Lahiri DK, Bosetti F, Greig NH, Rosi S. Tumor necrosis factor-alpha synthesis inhibitor 3,6’-dithiothalidomide attenuates markers of inflammation, Alzheimer pathology and behavioral deficits in animal models of neuroinflammation and Alzheimer’s disease. J Neuroinflammation. 2012;9:106. 10.1186/1742-2094-9-106. 22642825 10.1186/1742-2094-9-106 PMC3405480

[40] Gabbita SP, Srivastava MK, Eslami P, Johnson MF, Kobritz NK, Tweedie D, Greig NH, Zemlan FP, Sharma SP, Harris-White ME. Early intervention with a small molecule inhibitor for tumor necrosis factor-α prevents cognitive deficits in a triple transgenic mouse model of Alzheimer’s disease. J Neuroinflammation. 2012;9:99. 10.1186/1742. 22632257 10.1186/1742-2094-9-99 PMC3403851

[41] Chen ST, Hsu CY, Hogan EL, Maricq H, Balentine JD. A model of focal ischemic stroke in the rat: reproducible extensive cortical infarction. Stroke. 1986;17(4):738–43. 10.1161/01.STR.17.4.73. 2943059 10.1161/01.str.17.4.738

[42] Shen H, Luo Y, Kuo CC, Deng X, Chang CF, Harvey BK, Hoffer BJ, Wang Y. 9-Cis-retinoic acid reduces ischemic brain injury in rodents via bone morphogenetic protein. J Neurosci Res. 2009;87(2):545–55. 10.1002/jnr.21865. 18803283 10.1002/jnr.21865 PMC2628966

[43] Li Y, Perry TA, Kindy MS, Harvery BK, Tweedie D, Holloway HW, Powers K, ShenH, Egan JM, Sambamurti K, Bross A, Lahiri DK, Mattson MP, Hoffer BJ, Wang Y, Greig NH. GLP-1 receptor stimulation preserves primary cortical and dopaminergic neurons in cellular and rodent models of stroke and Parkinsonism. Proc Natl Acad Sci U S A 2009; 106(4): 1285–1290. 10.1073/pnas.0806720106 10.1073/pnas.0806720106 PMC2633544 19164583

[44] Borlongan CV, Hida H, Nishino H. Early assessment of motor dysfunctions aids in successful occlusion of the middle cerebral artery. NeuroReport. 1998;9(16):3615–21. 10.1097/00001756-199811160-00012. 9858369 10.1097/00001756-199811160-00012

[45] Borlongan CV, Sanberg PR. Elevated body swing test: a new behavioral parameter for rats with 6-hydroxydopamine-induced hemiparkinsonism. J Neurosci. 1995;15(7 Pt 2):5372–8. 10.1523/JNEUROSCI.15-07-05372.1995. 7623159 10.1523/JNEUROSCI.15-07-05372.1995 PMC6577895

[46] Charan J, Kantharia ND. How to calculate sample size in animal studies? J Pharmacol Pharmacother. 2013;4:303–6. 10.4103/0976-500X.119726. 24250214 10.4103/0976-500X.119726 PMC3826013

[47] Sampaio EP, Sarno EN, Galilly R, Cohn ZA, Kaplan G. Thalidomide selectively inhibits tumor necrosis factor alpha production by stimulated human monocytes. J Exp Med. 1991;173(3):699–703. 10.1084/jem.173.3.699. 1997652 10.1084/jem.173.3.699 PMC2118820

[48] Obach RS. Prediction of human clearance of twenty-nine drugs from hepatic microsomal intrinsic clearance data: an examination of in vitro half-life approach and nonspecific binding to microsomes. Drug Metab Dispos. 1999;27:1350–9. 10534321

[49] Liu Y, Mo CC, Hartley-Brown MA, Sperling AS, Midha S, Yee AJ, Bianchi G, Piper C, Tattersall A, Nadeem O, Laubach JP, Richardson PG. Targeting Ikaros and Aiolos: reviewing novel protein degraders for the treatment of multiple myeloma, with a focus on iberdomide and mezigdomide. Expert Rev Hematol. 2024;17:445–65. 10.1080/17474086.2024.2382897. 39054911 10.1080/17474086.2024.2382897

[50] Nutt MJ, Stewart SG. Strengthening molecular glues: design strategies for improving thalidomide analogs as cereblon effectors and anticancer agents. Drug Discov Today. 2024;29:104010. 10.1016/j.drudis.2024.104010. 38704021 10.1016/j.drudis.2024.104010

[51] Chamberlain PP, Lopez-Girona A, Miller K, Carmel G, Pagarigan B, Chie-Leon B, Rychak E, Corral LG, Ren YJ, Wang M, Riley M, Delker SL, Ito T, Ando H, Mori T, Hirano Y, Handa H, Hakoshima T, Daniel TO, Cathers BE. Structure of the human Cereblon-DDB1-lenalidomide complex reveals basis for responsiveness to thalidomide analogs. Nat Struct Mol Biol. 2014;21(9):803–9. 10.1038/nsmb.2874. 25108355 10.1038/nsmb.2874

[52] Lee H, Neri P, Bahlis NJ. Cereblon-targeting ligase degraders in myeloma: mechanisms of action and resistance. Hematol Oncol Clin North Am. 2024;38(2):305–19. 10.1016/j.hoc.2024.01.001. 38302306 10.1016/j.hoc.2024.01.001

[53] Ito T, Ando H, Suzuki T, Ogura T, Hotta K, Imamura Y, Yamaguchi Y, Handa H. Identification of a primary target of thalidomide teratogenicity. Science. 2010;327:1345–50. 10.1126/science.1177319. 20223979 10.1126/science.1177319

[54] Fischer ES, Böhm K, Lydeard JR, Yang H, Stadler MB, Cavadini S, Nagel J, Serluca F, Acker V, Lingaraju GM, Tichkule RB, Schebesta M, Forrester WC, Schirle M, Hassiepen U, Ottl J, Hild M, Beckwith RE, Harper JW, Jenkins JL, Thomä NH. Structure of the DDB1-CRBN E3 ubiquitin ligase in complex with thalidomide. Nature. 2014;512:49–53. 10.1038/nature13527. 25043012 10.1038/nature13527 PMC4423819

[55] Chamberlain PP, Lopez-Girona A, Miller K, Carmel G, Pagarigan B, Chie-Leon B, Rychak E, Corral LG, Ren YJ, Wang M, Riley M, Delker SL, Ito T, Ando H, Mori T, Hirano Y, Handa H, Hakoshima T, Daniel TO, Cathers BE. Structure of the human Cereblon-DDB1-lenalidomide complex reveals basis for responsiveness to thalidomide analogs. Nat Struct Mol Biol. 2014;21:803–9. 10.1038/nsmb.2874. 25108355 10.1038/nsmb.2874

[56] Mori T, Ito T, Liu S, Ando H, Sakamoto S, Yamaguchi Y, Tokunaga E, Shibata N, Handa H, Hakoshima T. Structural basis of thalidomide enantiomer binding to cereblon. Sci Rep. 2018;8:1294. 10.1038/s41598-018-19202-7. 29358579 10.1038/s41598-018-19202-7 PMC5778007

[57] Chesi M, Matthews GM, Garbitt VM, Palmer SE, Shortt J, Lefebure M, Stewart AK, Johnstone RW, Bergsagel PL. Drug response in a genetically engineered mouse model of multiple myeloma is predictive of clinical efficacy. Blood. 2012;120:376–85. 10.1182/blood-2012-02-412783. 22451422 10.1182/blood-2012-02-412783 PMC3398763

[58] Krönke J, Fink EC, Hollenbach PW, MacBeth KJ, Hurst SN, Udeshi ND, Chamberlain PP, Mani DR, Man HW, Gandhi AK, Svinkina T, Schneider RK, McConkey M, Järås M, Griffiths E, Wetzler M, Bullinger L, Cathers BE, Carr SA, Chopra R, Ebert BL. Lenalidomide induces ubiquitination and degradation of CK1α in del(5q) MDS. Nature. 2015;523:183–8. 10.1038/nature14610. 26131937 10.1038/nature14610 PMC4853910

[59] Gemechu Y, Millrine D, Hashimoto S, Prakash J, Sanchenkova K, Metwally H, Gyanu P, Kang S, Kishimoto T. Humanized cereblon mice revealed two distinct therapeutic pathways of immunomodulatory drugs. Proc Natl Acad Sci U S A. 2018;115(46):11802–7. 10.1073/pnas. 30373817 10.1073/pnas.1814446115 PMC6243262

[60] Millrine D, Miyata H, Tei M, Dubey P, Nyati K, Nakahama T, Gemechu Y, Ripley B, Kishimoto T. Immunomodulatory drugs inhibit TLR4-induced type-1 interferon production independently of Cereblon via suppression of the TRIF/IRF3 pathway. Int Immunol. 2016;28(6):307–15. 10.1093/intimm/dxw005. 26865412 10.1093/intimm/dxw005

[61] Millrine D, Kishimoto T. A brighter side to thalidomide: its potential use in immunological disorders. Trends Mol Med. 2017;23:348–61. 10.1016/j.molmed.2017.02.006. 28285807 10.1016/j.molmed.2017.02.006

[62] Lecca D, Hsueh SC, Luo W, Tweedie D, Kim DS, Baig AM, Vargesson N, Kim YK, Hwang I, Kim S, Hoffer BJ, Chiang YH, Greig NH. Novel, thalidomide-like, non-cereblon binding drug tetrafluorobornylphthalimide mitigates inflammation and brain injury. J Biomed Sci. 2023;30:16. 10.1186/s12929-023-00907-5. 36872339 10.1186/s12929-023-00907-5 PMC9987061

[63] Moreira AL, Sampaio EP, Zmuidzinas A, Frindt P, Smith KA, Kaplan G. Thalidomide exerts its inhibitory action on tumor necrosis factor alpha by enhancing mRNA degradation. J Exp Med. 1993;177:1675–80. 10.1084/jem.177.6.1675. 8496685 10.1084/jem.177.6.1675 PMC2191046

[64] Bartlett JB, Dredge K, Dalgleish AG. The evolution of thalidomide and its IMiD derivatives as anticancer agents. Nat Rev Cancer. 2004;4:314. 15057291 10.1038/nrc1323

[65] Yagyu T, Kobayashi H, Matsuzaki H, Wakahara K, Kondo T, Kurita N, Sekino H, Inagaki K, Suzuki M, Kanayama N, Terao T. Thalidomide inhibits tumor necrosis factor-alpha-induced interleukin-8 expression in endometriotic stromal cells, possibly through suppression of nuclear factor-kappaB activation. J Clin Endocrinol Metab. 2005;90:3017–21. 10.1210/jc.2004-1946. 15687330 10.1210/jc.2004-1946

[66] Keifer JA, Guttridge DC, Ashburner BP, Baldwin AS. Jr Inhibition of NF-kappa B activity by thalidomide through suppression of IkappaB kinase activity. J Biol Chem. 2001;276:22382–7. 10.1074/jbc.M100938200. 11297551 10.1074/jbc.M100938200

[67] Palmer AA, Stezoski JP, Janesko-Feldman K, Kochanek PM, Drabek T. Targeting TNFα-mediated cytotoxicity using thalidomide after experimental cardiac arrest in rats: an exploratory study. Exp Ther Med. 2022;23:380. 10.3892/etm.2022.11307. 35495588 10.3892/etm.2022.11307 PMC9019692

[68] Zamyatina A, Heine H. Lipopolysaccharide recognition in the crossroads of TLR4 and caspase-4/11 mediated inflammatory pathways. Front Immunol. 2020;11:585146. 10.3389/fimmu.2020.585146. 33329561 10.3389/fimmu.2020.585146 PMC7732686

[69] Arslan F, Keogh B, McGuirk P, Parker AE. TLR2 and TLR4 in ischemia reperfusion injury. Mediators Inflamm. 2010;2010:704202. 10.1155/2010/704202. 20628516 10.1155/2010/704202 PMC2902053

[70] Min Y, Wi SM, Kang JA, Yang T, Park CS, Park SG, Chung S, Shim JH, Chun E, Lee KY. Cereblon negatively regulates TLR4 signaling through the attenuation of ubiquitination of TRAF6. Cell Death Disease. 2016;7(7):e2313. 10.1038/cddis.2016.226. 27468689 10.1038/cddis.2016.226 PMC4973362

[71] Zhou L, Hao Z, Wang G, Xu G. Cereblon suppresses the formation of pathogenic protein aggregates in a p62-dependent manner. Human Molecul Genet. 2018;27(4):667–78. 10.1093/hmg/ddx433. 10.1093/hmg/ddx433 29272390

[72] Kontoyiannis D, Pasparakis M, Pizarro TT, Cominelli F, Kollias G. Impaired on/off regulation of TNF biosynthesis in mice lacking TNF AU-rich elements: implications for joint and gut-associated immunopathologies. Immunity. 1999;10:387–98. 10.1016/s1074-7613(00)80038-2. 10204494 10.1016/s1074-7613(00)80038-2

[73] Asirvatham AJ, Magner WJ, Tomasi TB. miRNA regulation of cytokine genes. Cytokine. 2009;45:58–69. 10.1016/j.cyto.2008.11.010. 19121586 10.1016/j.cyto.2008.11.010 PMC3129852

[74] Hayashi T, Hideshima T, Akiyama M, Podar K, Yasui H, Raje N, Kumar S, Chauhan D, Treon SP, Richardson P, Anderson KC. Molecular mechanisms whereby immunomodulatory drugs activate natural killer cells: clinical application. Br J Haematol. 2005;128:192–203. 10.1111/j.1365-2141.2004.05286.x. 15638853 10.1111/j.1365-2141.2004.05286.x

[75] Tuttolomondo A, Pecoraro R, Pinto A. Studies of selective TNF inhibitors in the treatment of brain injury from stroke and trauma: a review of the evidence to date. Drug Des Devel Ther. 2014;8:2221–38. 10.1111/j.1365-2141.2004.05286.x. 25422582 10.2147/DDDT.S67655 PMC4232043

[76] Liesz A, Suri-Payer E, Veltkamp C, Doerr H, Sommer C, Rivest S, Giese T, Veltkamp R. Regulatory T cells are key cerebroprotective immunomodulators in acute experimental stroke. Nat Med. 2009;15(2):192–9. 10.1038/nm.1927. 19169263 10.1038/nm.1927

[77] Zhu H, Hu S, Li Y, Sun Y, Xiong X, Hu X, Chen J, Qiu S. Interleukins and ischemic stroke Front Immunol. 2022;13:828447. 10.3389/fimmu.2022.828447. 35173738 10.3389/fimmu.2022.828447 PMC8841354

[78] Sun W, Wang S, Nan S. The prognostic determinant of interleukin-10 in patients with acute ischemic stroke: an analysis from the perspective of disease management. Dis Markers. 2021;2021:6423244. 10.1155/2021/6423244. 34336007 10.1155/2021/6423244 PMC8313368

[79] Bonaventura A, Liberale L, Vecchié A, Casula M, Carbone F, Dallegri F, Montecucco F. Update on inflammatory biomarkers and treatments in ischemic stroke. Int J Mol Sci. 2016;17:1967. 10.3390/ijms17121967. 27898011 10.3390/ijms17121967 PMC5187767

[80] European Medicines Agency. Assessment Report: Pomalidomide Celgene [Internet]. 2013. [Accessed 02/10/2025]. https://www.ema.europa.eu/documents/assessment-report/pomalidomide-celgene-epar-public-assessment-report_en.pdf

[81] Nam YH, Brensinger CM, Bilker WB, Leonard CE, Kasner SE, Grosser T, Li X, Hennessy S. Nonsteroidal anti-inflammatory drug choice and adverse outcomes in clopidogrel users: a retrospective cohort study. PLoS ONE. 2018;13(3):e0193800. 10.1371/journal.pone.0193800. 29538453 10.1371/journal.pone.0193800 PMC5851628

[82] Jiang Y, Wang J, Rozewski DM, Kolli S, Wu CH, Chen CS, Yang X, Hofmeister CC, Byrd JC, Johnson AJ, Phelps MA. Sensitive liquid chromatography/mass spectrometry methods for quantification of pomalidomide in mouse plasma and brain tissue. J Pharm Biomed Anal. 2014;88:262–8. 10.1016/j.jpba.2013.08.036. 24095801 10.1016/j.jpba.2013.08.036 PMC3860284

[83] Keller K, Geyer M, Munzel T, Ostad MA. Gender-differences in prevalence and outcome of ischemic stroke and promoting factors of atrial thrombi. Artery Res. 2018;22:68–78. 10.1016/j.artres.2018.05.004.

[84] Roy-O’Reilly M, McCullough LD. Age and sex are critical factors in ischemic stroke pathology. Endocrinology. 2018;159:3120–31. 10.1210/en.2018-00465. 30010821 10.1210/en.2018-00465 PMC6963709

[85] Tariq MB, Lee J, McCullough LD. Sex differences in the inflammatory response to stroke. Semin Immunopathol. 2023;45:295–313. 10.1007/s00281-022-00969-x. 36355204 10.1007/s00281-022-00969-x PMC10924671

[86] Zhong X, Sun Y, Lu Y, Xu L. Immunomodulatory role of estrogen in ischemic stroke: neuroinflammation and effect of sex. Front Immunol. 2023;14:1164258. 10.3389/fimmu.2023.1164258. 37180115 10.3389/fimmu.2023.1164258 PMC10167039

[87] Spittau B. Aging microglia—phenotypes, functions and implications for age-related neurodegenerative diseases. Front Aging Neurosci. 2017;9:194. 10.3389/fnagi.2017.00194. 28659790 10.3389/fnagi.2017.00194 PMC5469878

[88] Fulop T, Larbi A, Dupuis G, Le Page A, Frost EH, Cohen AA, Witkowski JM, Franceschi C. Immunosenescence and inflammaging as two sides of the same coin: friends or foes? Front Immunol. 2018;8:1960. 10.3389/fimmu.2017.01960. 29375577 10.3389/fimmu.2017.01960 PMC5767595

[89] Andronie-Cioara FL, Ardelean AI, Nistor-Cseppento CD, Jurcau A, Jurcau MC, Pascalau N, Marcu F. Molecular mechanisms of neuroinflammation in aging and Alzheimer’s disease progressioN. Int J Mol Sci. 2023;24:1869. 10.3390/ijms24031869. 36768235 10.3390/ijms24031869 PMC9915182

