# Supplementary material for: 3-Monothiopomalidomide, a new immunomodulatory imide drug (IMiD), blunts inflammation and mitigates ischemic stroke in the rat
Source: GeroScience. 2025 Mar 17;47(4):5763–83. doi: 10.1007/s11357-025-01573-1 (PMC12397098; doi:10.1007/s11357-025-01573-1)

**3-Monothiopomalidomide, a new immunomodulatory imide drug (IMiD), blunts inflammation and mitigates ischemic stroke in the rat.**

**Supplemental Information**

**Nanoformulation Preparation for LC/MS Studies:** A sample of 3-MP (35.27 mg) and Tween-80 (37.5 µl) was combined with water (4927.5 µl) in a glass vial containing ~ 200 mg of 0.1-0.2 mm yttrium-stabilized zirconia-silica beads (Silibeads, Typ ZY Sigmund Linder, Germany). This ‘bulk’ preparation was vortexed for 10 min at 2500 rpm. After this the ‘bulk’ preparation was transferred to 1.5 ml Eppendorf tubes containing the Silibeads and oscillated at 3000 rpm for two periods of 45 min at 4^o^C, using a Disrupter Genie, Scientific Industries, USA. After the second vortex period the nanoformulation was passed through a sterile filter (40 µm, Falcon Ref# 352340) under aseptic conditions. The filtrate was collected and transferred to a new glass vial and stored at 4^o^C until samples were required for LC/MS assessment.

**Nanoformulation LC/MS Assays:** A rudimentary assay was devised to assess the relative stability of nanoformulated 3-MP.  In short, a sample of nanoformulated 3-MP was monitored for degradation until, roughly, 10% of the original material was lost due to hydrolysis and other such factors. In this regard, 3-MP was nanoformulated (as described above) in Tween 80/water to an approximate concentration of 24mM and placed into a standard glass vial, and this heterogeneous suspension served as the bulk stock for all further stability experiments, with t=0 starting immediately after the formulation was completed.   The bulk stock was allowed to stand in the dark at 4^o^C over the course of approximately 100 days, and portions were removed periodically for LC/MS analysis.  In this regard, at defined time points, aliquots of the stock were further diluted into a 50/50 mixture of LC/MS-grade Acetonitrile/Water (without acid) to a concentration of roughly 240 µM, yielding homogenous solutions after a brief period of shaking.  These samples were immediately analyzed.  The first series of analyses were conducted almost daily until 14 days, at which point the analyses were conducted once a week thereafter. The areas of the ensuing UV peaks detected at 254nm served as the basis for analysis, and the stock nanoformulation was monitored as a function of derivation from the 3-MP nanoformulation t=0 area (defined as “100% Area Percent” in the initial trace).  While small fluctuations and presumed breakdown products were first selected and integrated by the OpenLab ChemStation software at t=28 days, it was not until t=49 days that these peaks were consistently detected (primarily via suspected hydrolysis to pomalidomide based upon retention time and m/z).   From the period of t=49 days until t=91 days, this suspected degradation remained largely consistent.  At t=98 days, the area of the 3-MP peak finally fell below 90% relative intensity, and the assay was stopped.

3-MP, non-nanoformulated primary material:


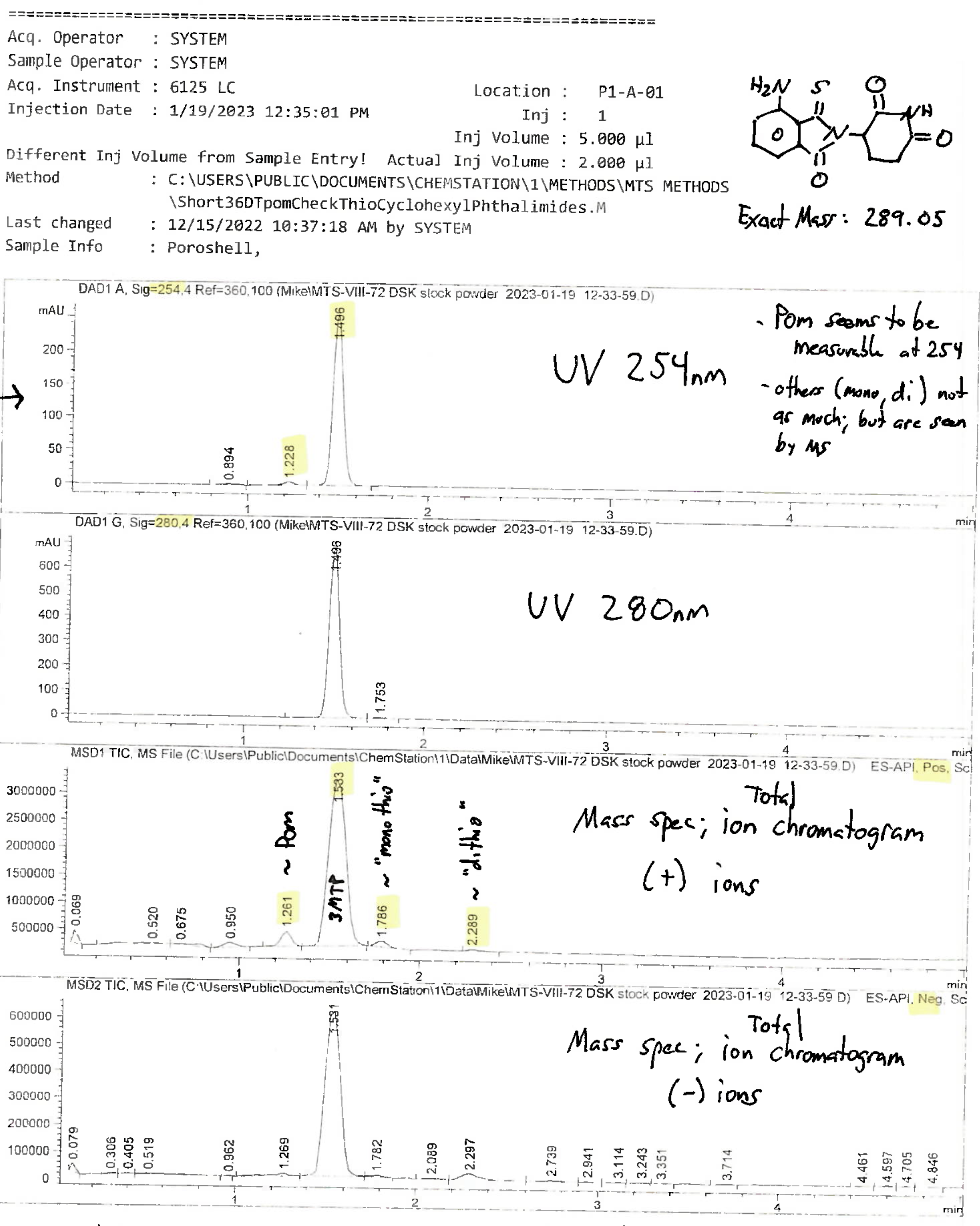


Nanoformulated samples

t=0


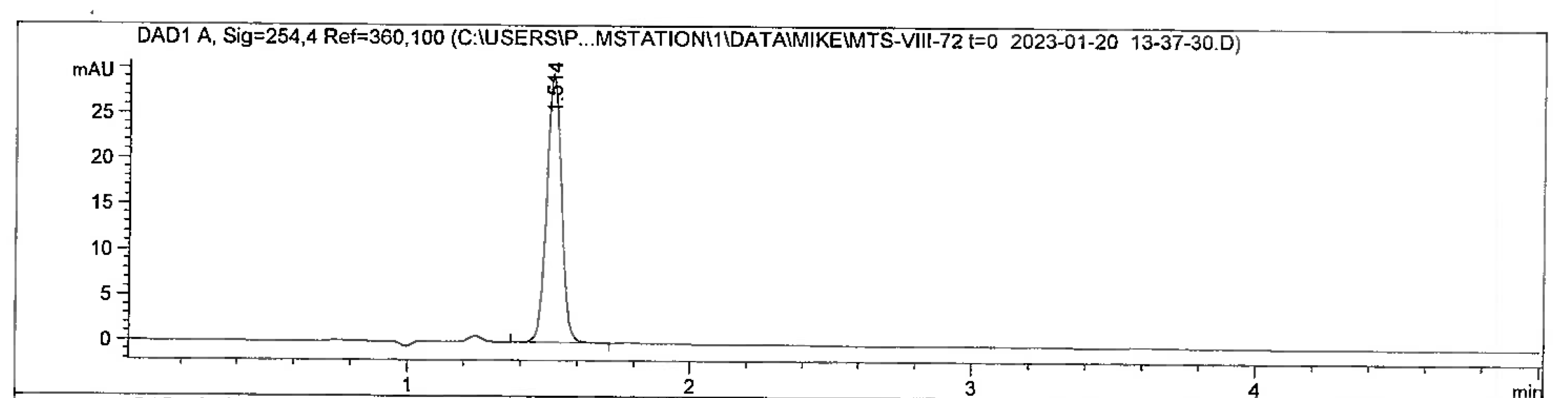


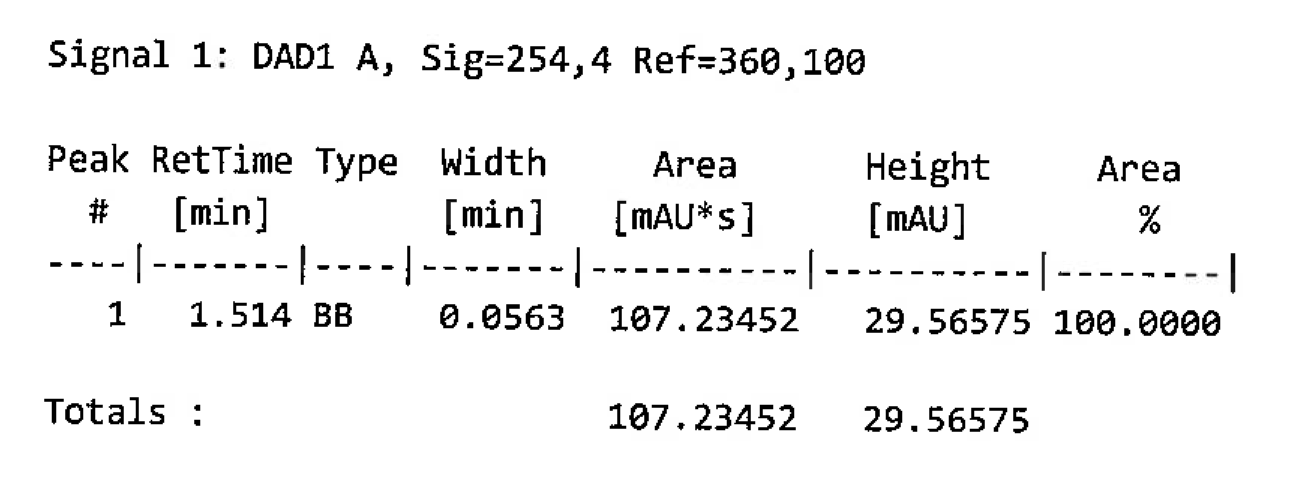


t=5 days


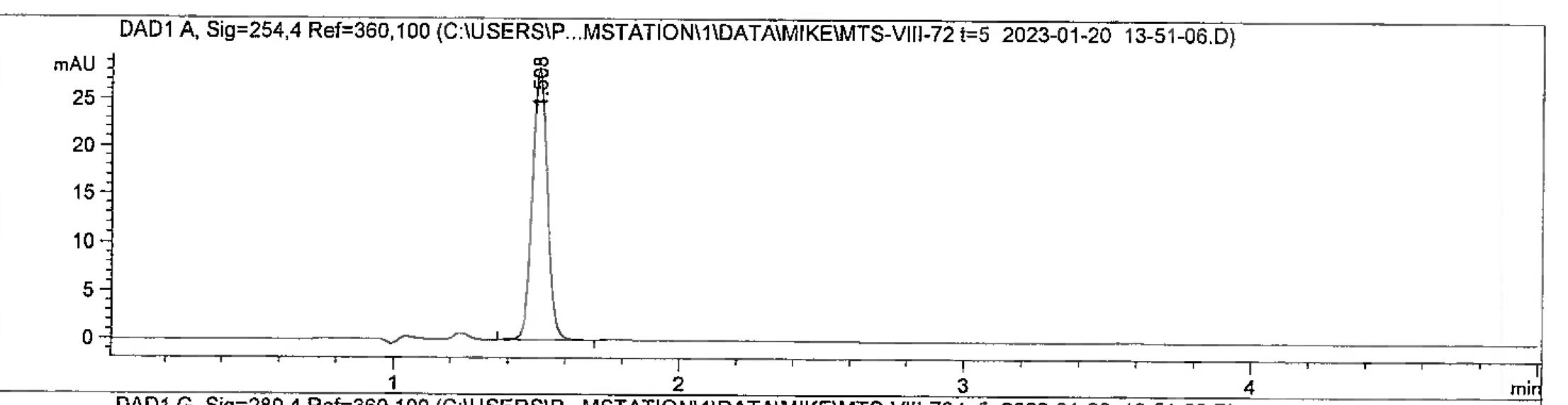


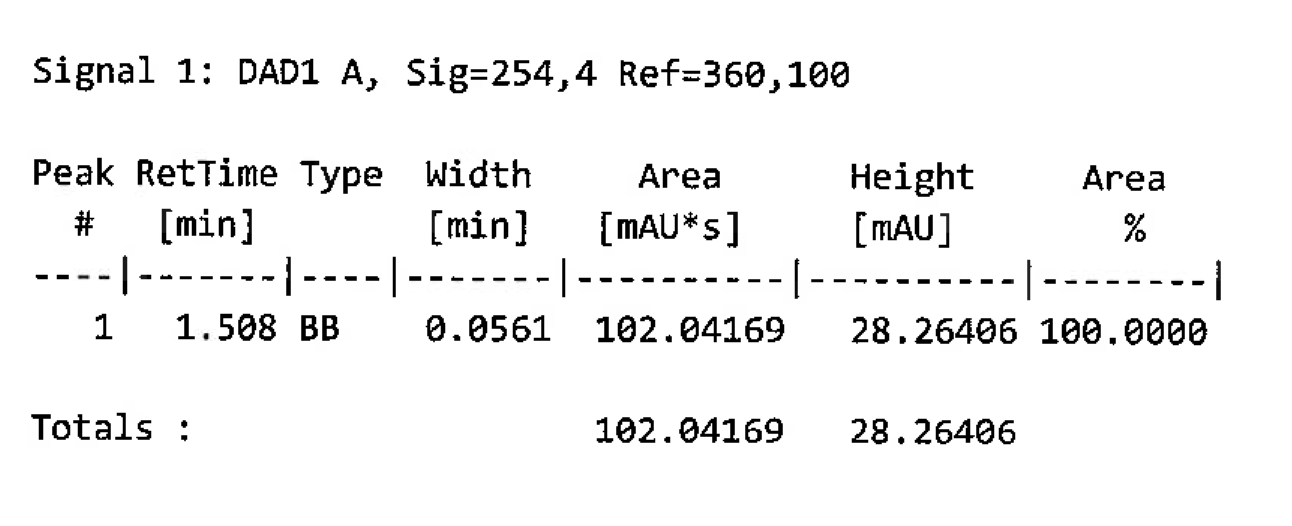


t=10 days


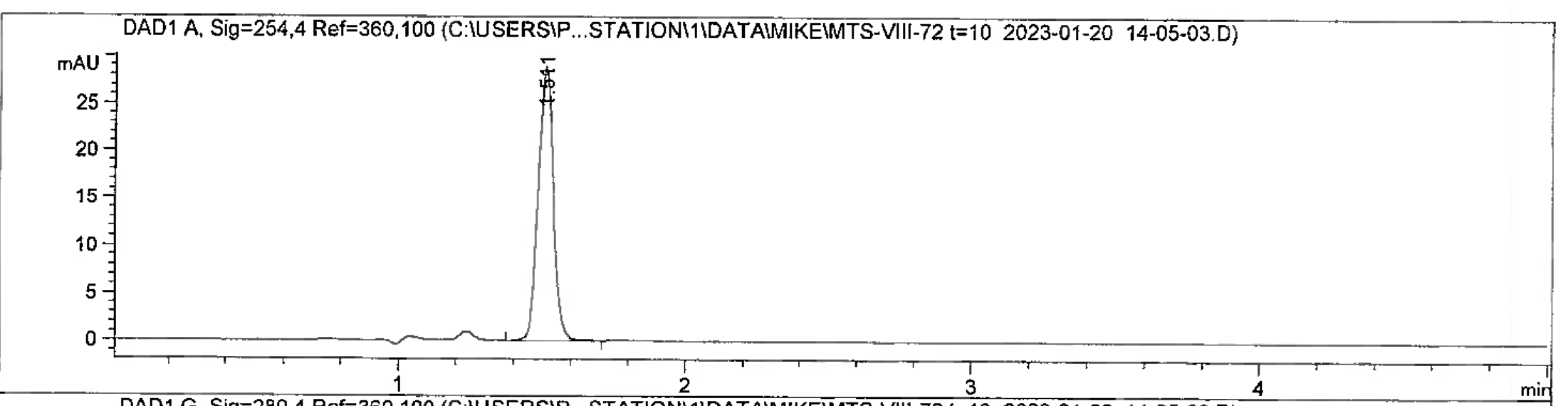


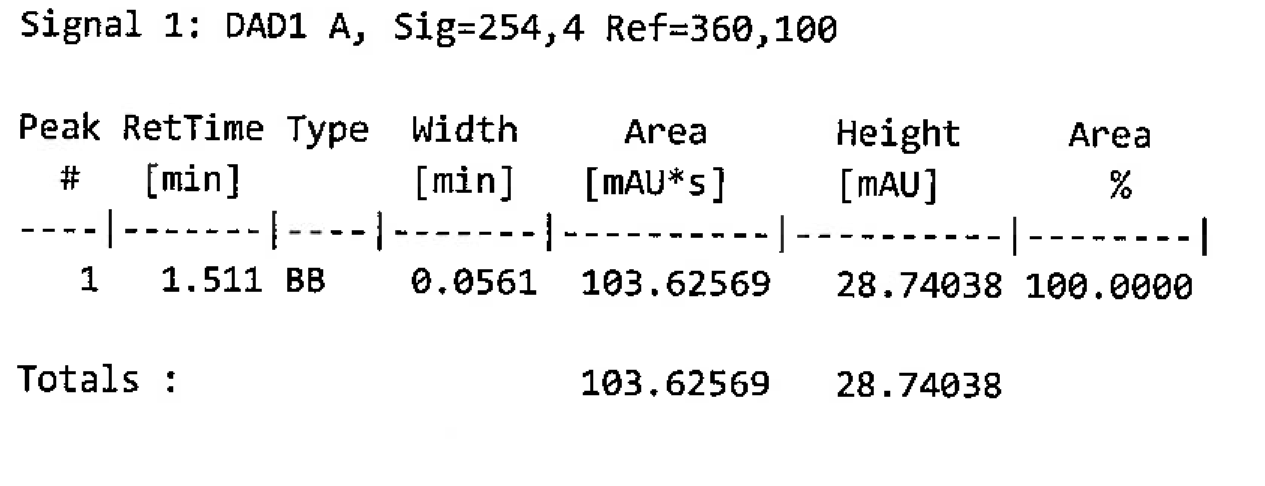


t=28 days


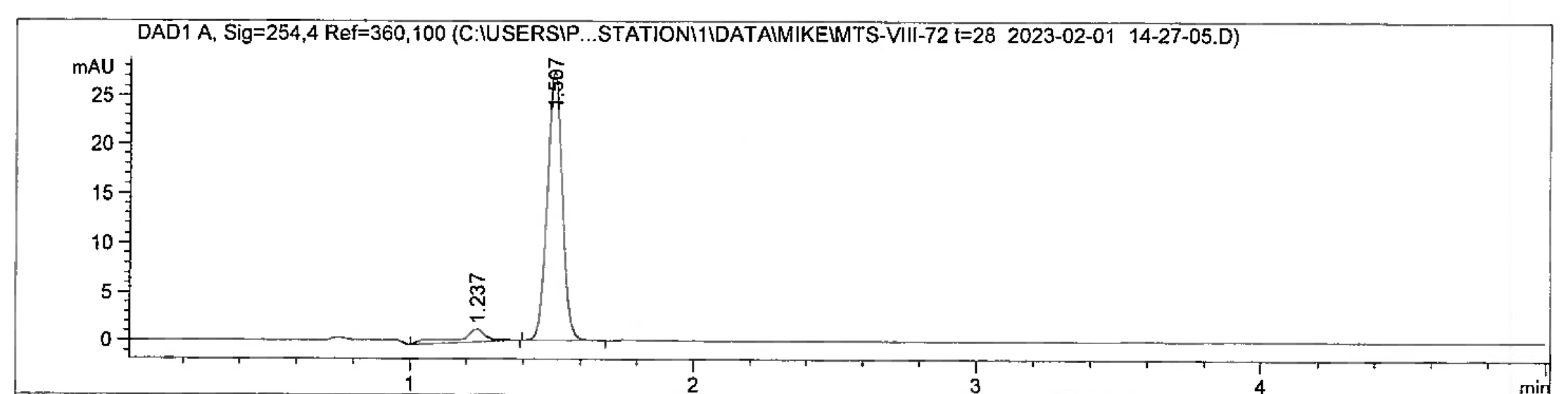


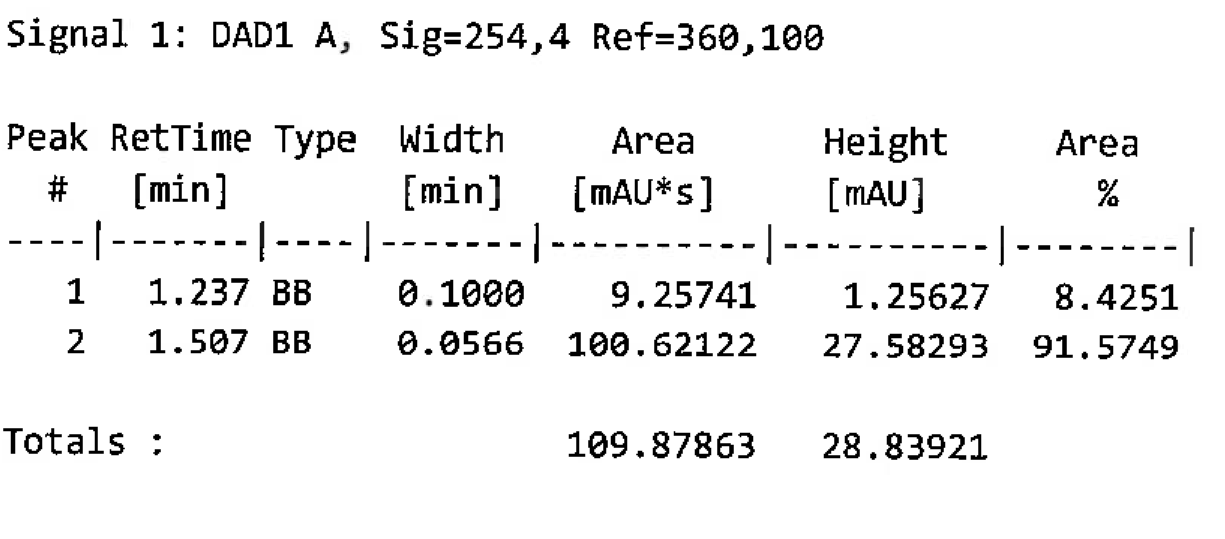


t=49 days


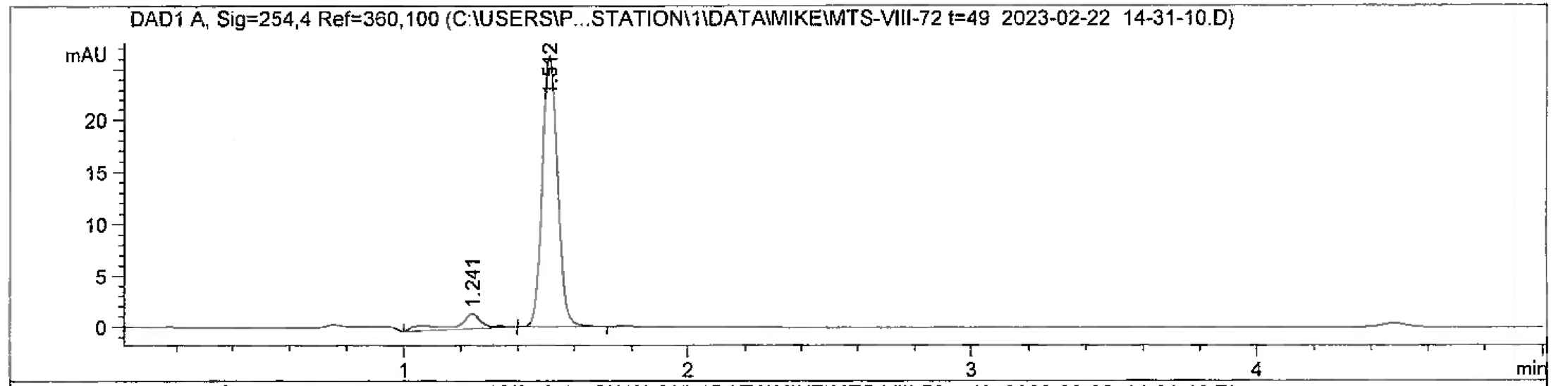


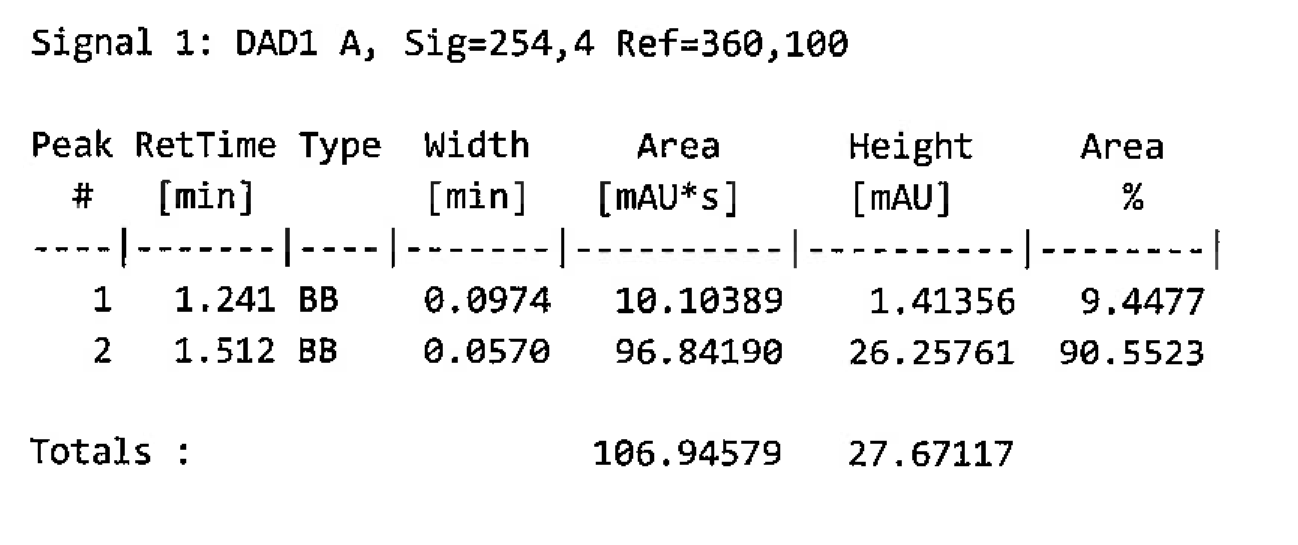


t=56 days


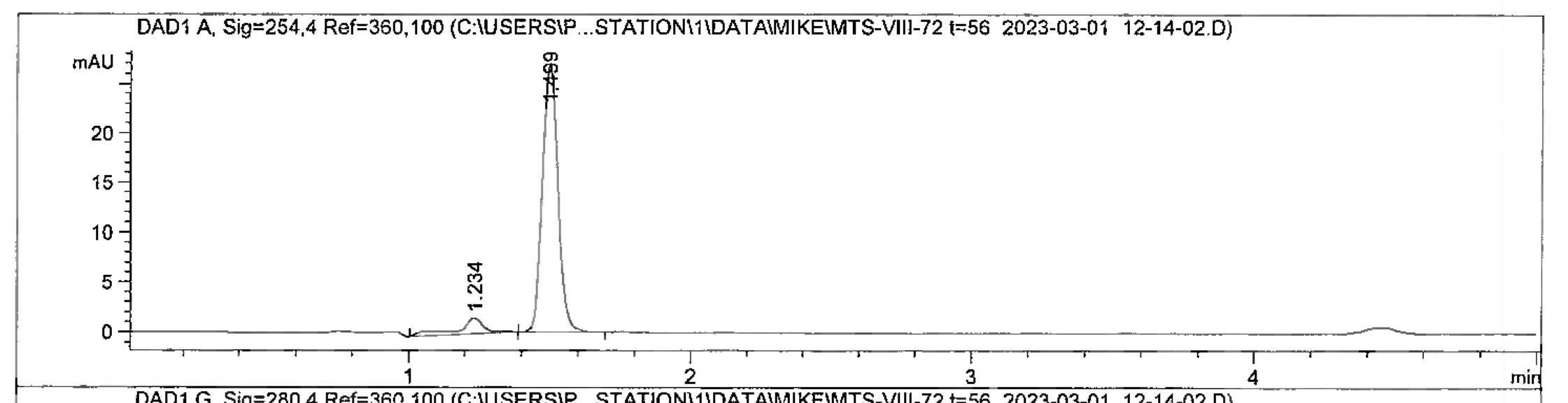


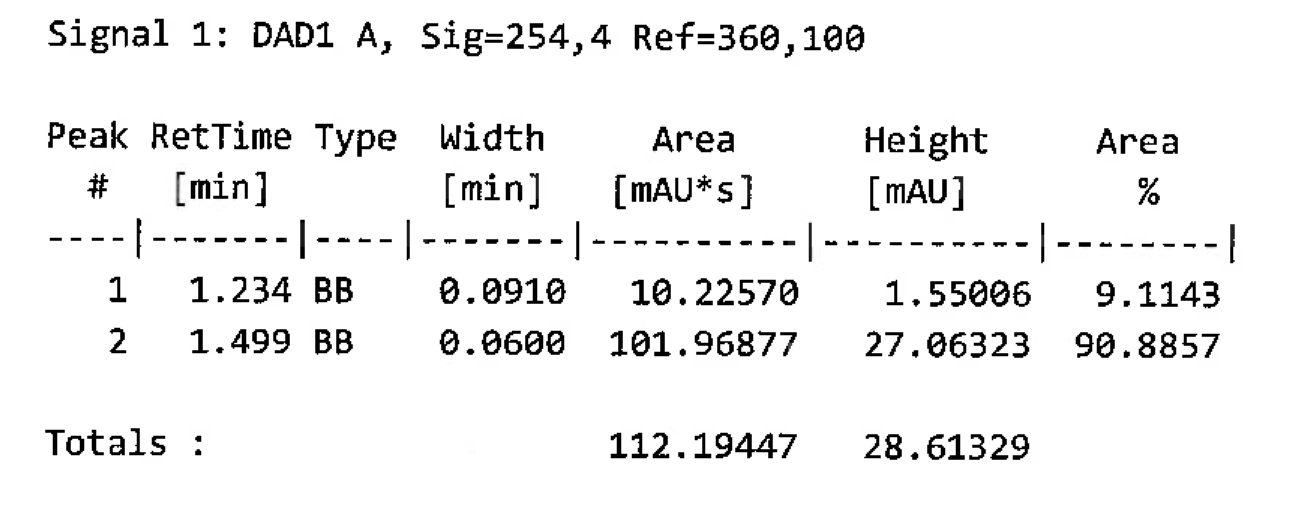


t=91 days


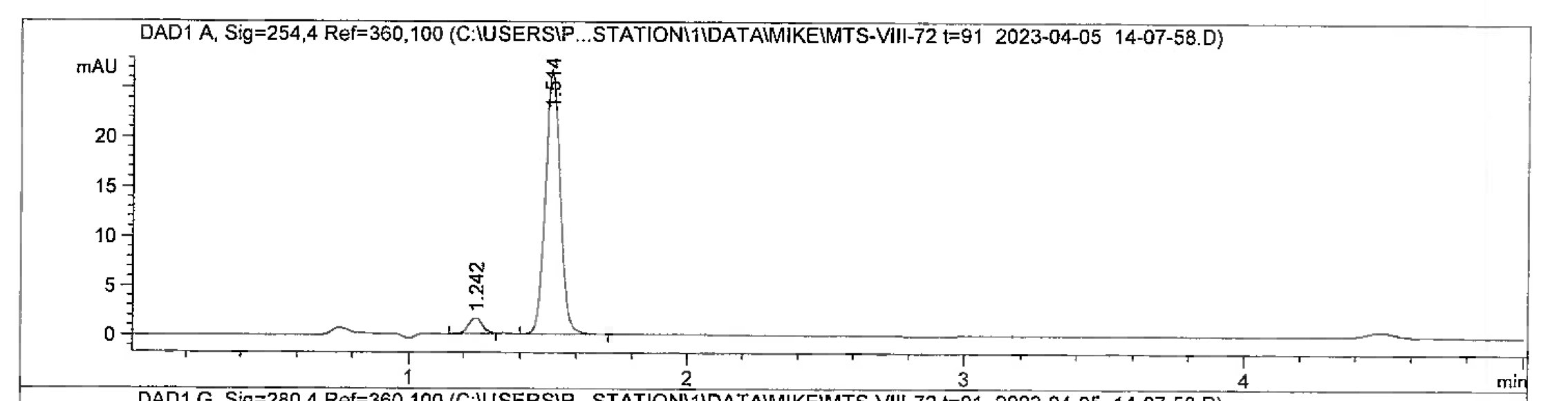


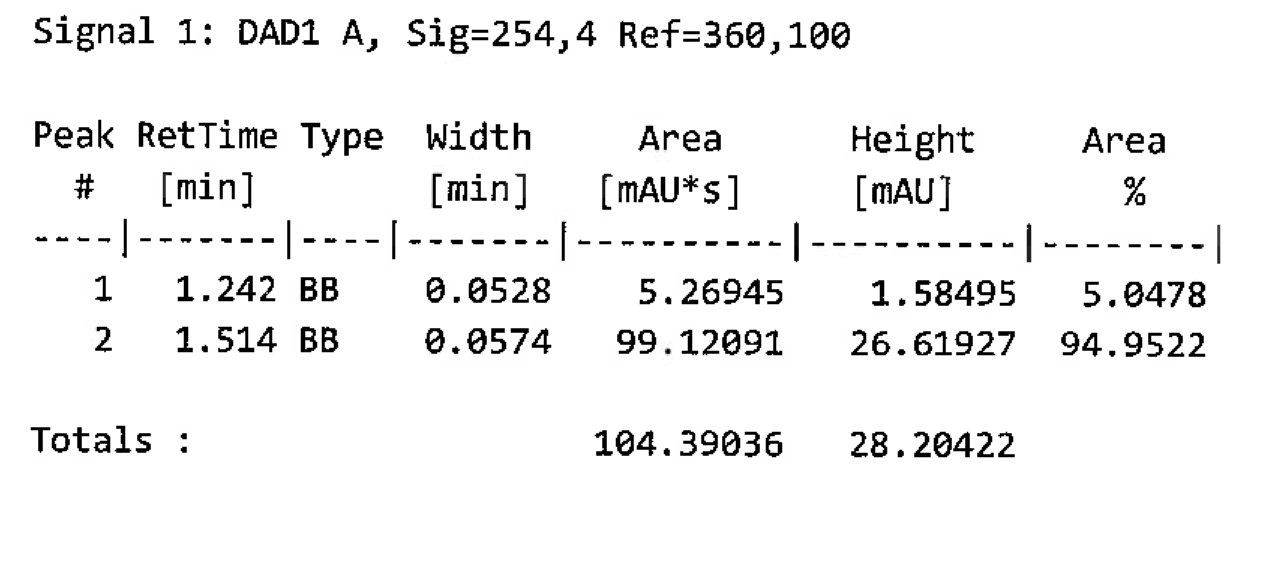


t=98 days


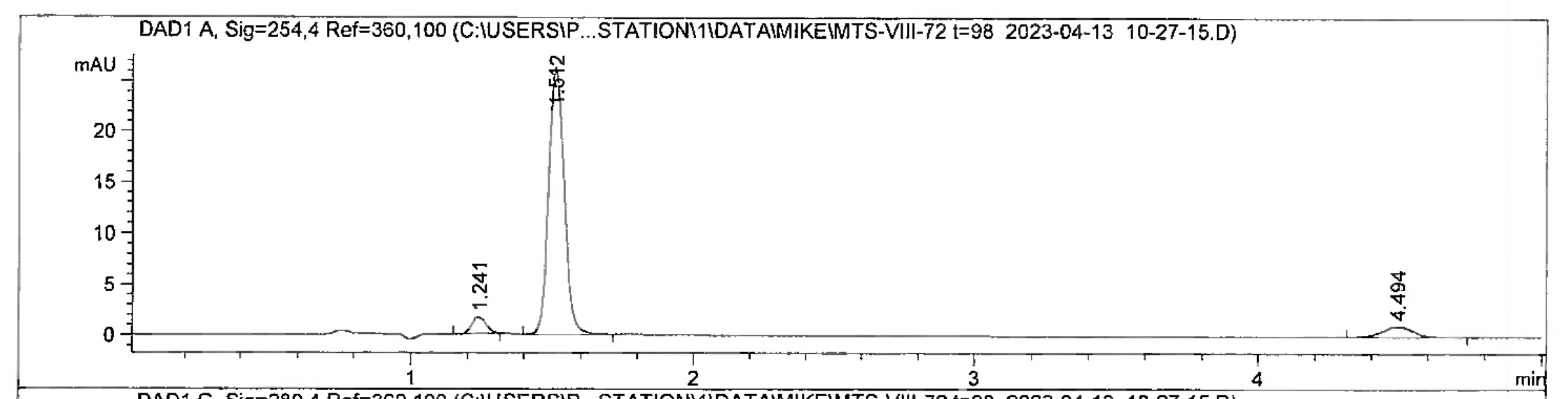


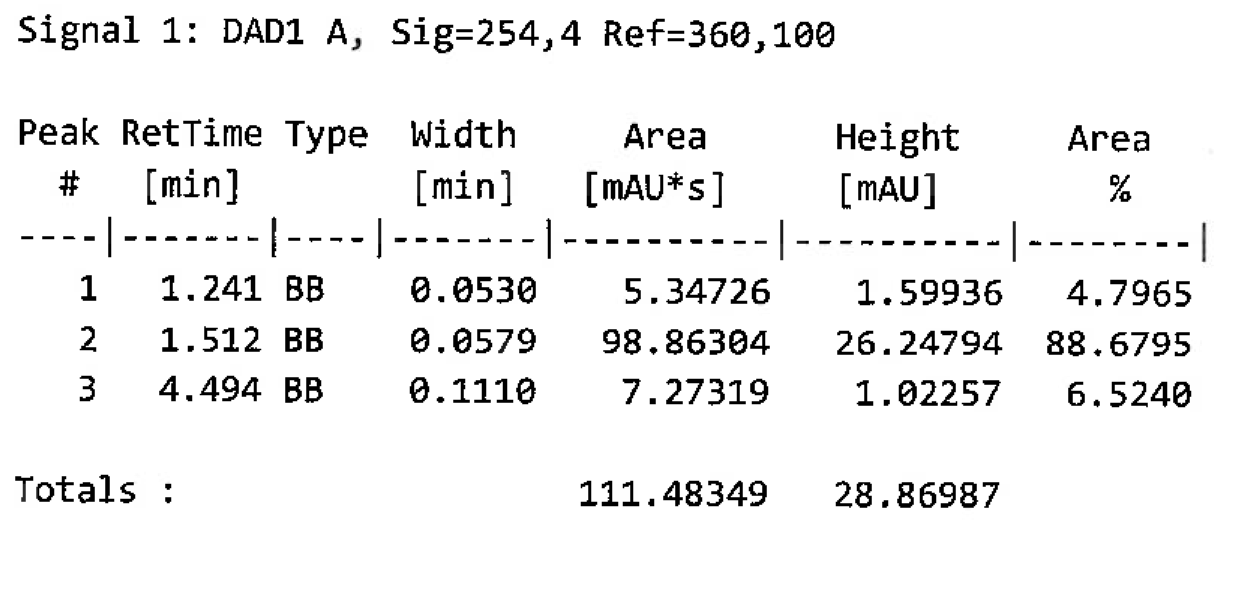

Supplement: Supplementary file 1 — Supplementary file1 (DOCX 2035 KB) [file 11357_2025_1573_MOESM1_ESM.docx]
